# Supplementary figures and images for: Identifying the drivers of computationally detected correlated evolution among sites under antibiotic selection
Source: Evol Appl. 2020 Feb 13;13(4):781–93. doi: 10.1111/eva.12900 (PMC7086105; doi:10.1111/eva.12900)

Observed

Expected

Was Mutation in a Focal Gene?

(a)

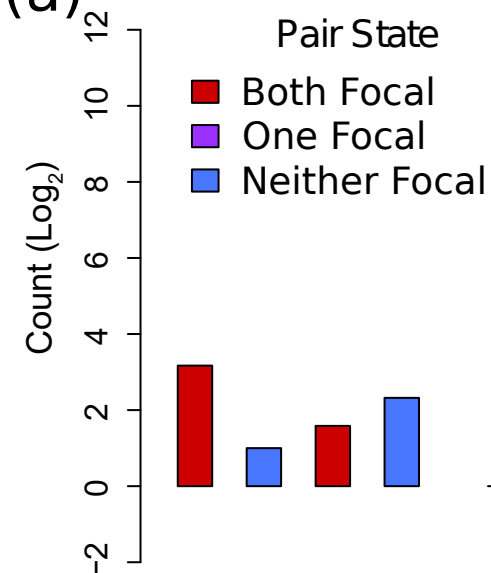

(b)

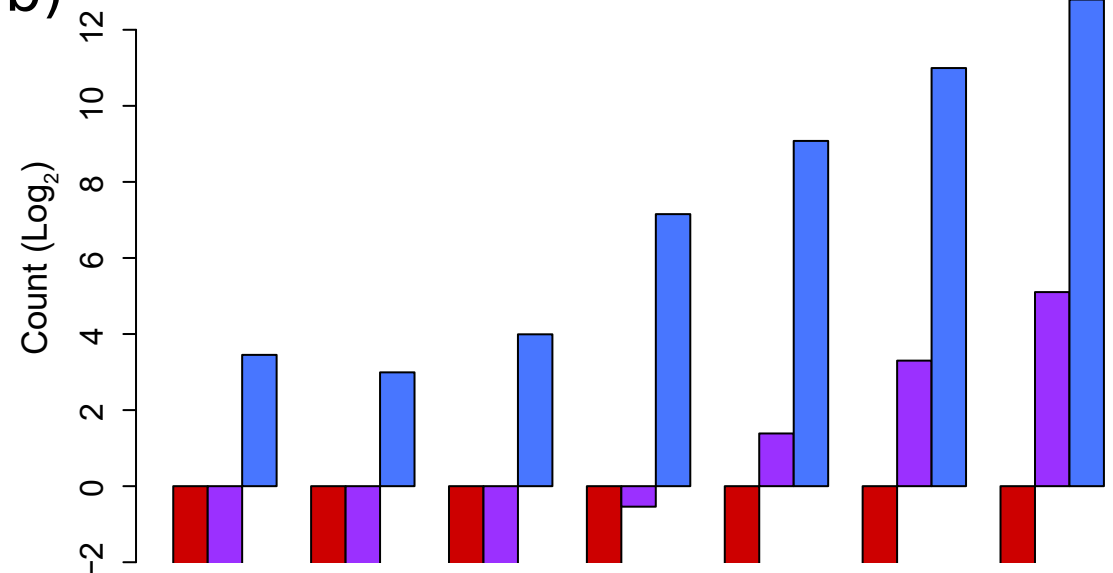

Type of Mutation

(c)

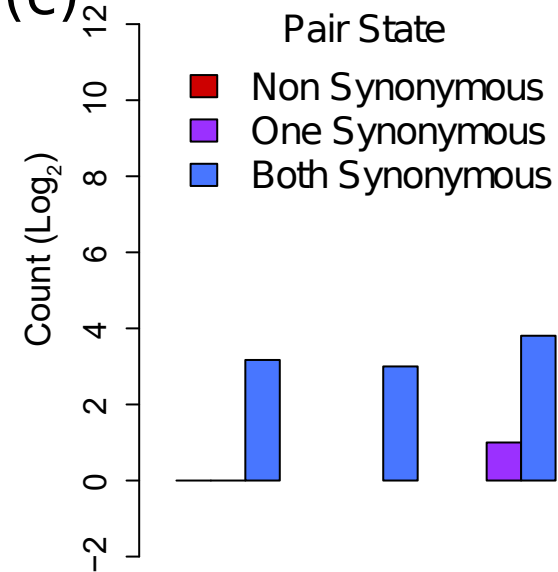

(d)

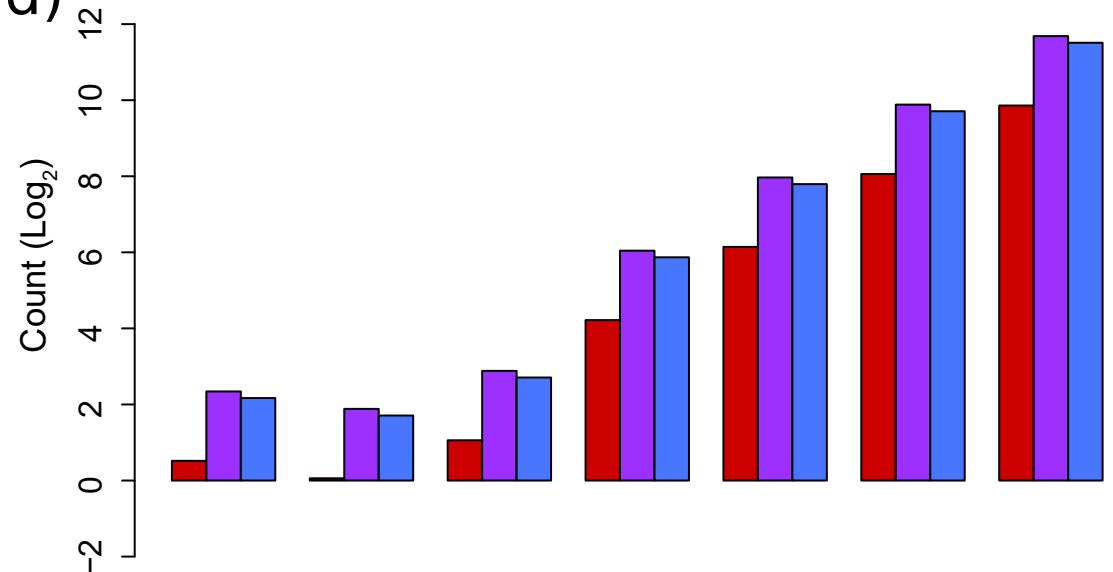

Is the Pair Intragenic?

(e)

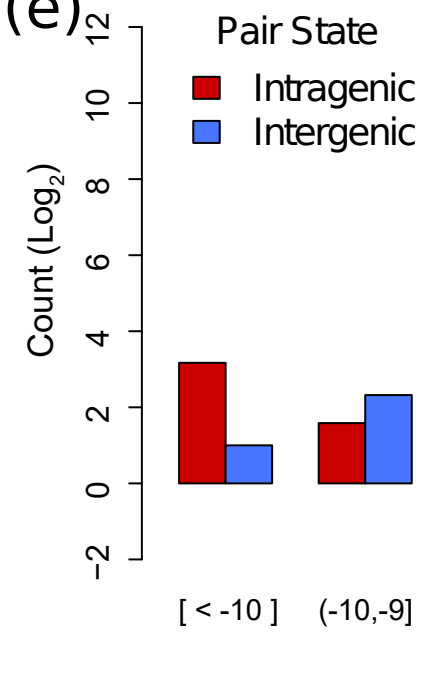

(f)

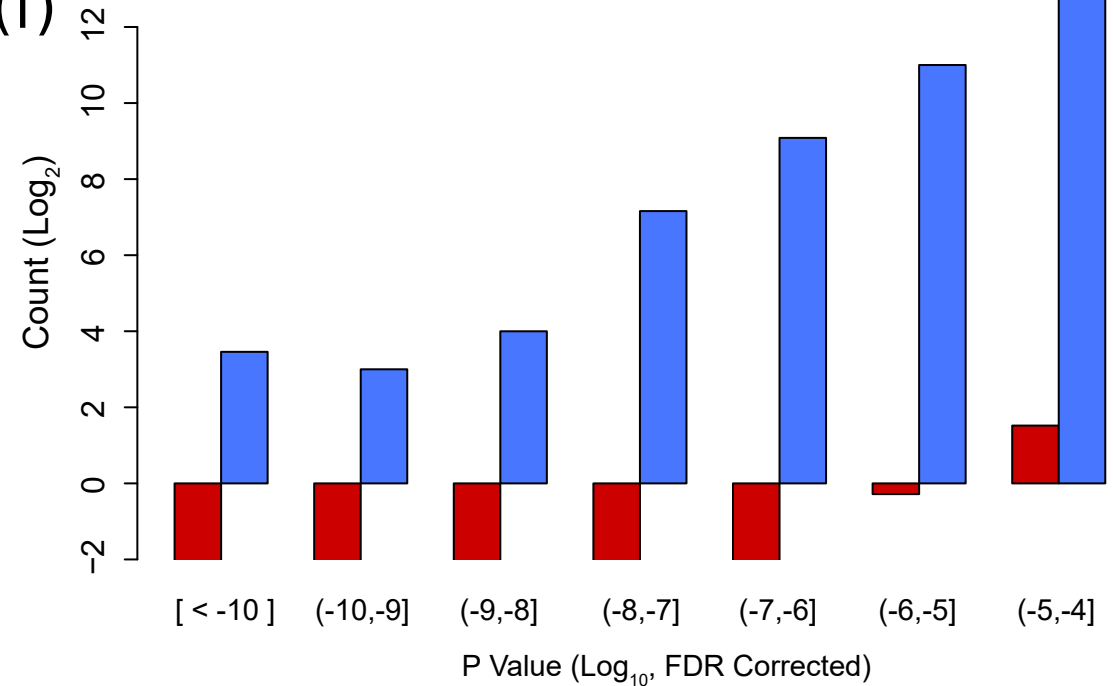

Supplement: Supplementary file 1 [file EVA-13-781-s001.pdf]

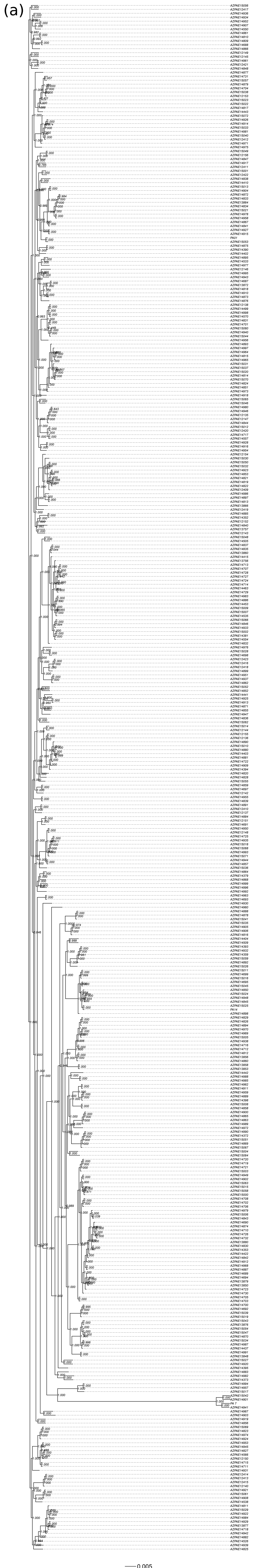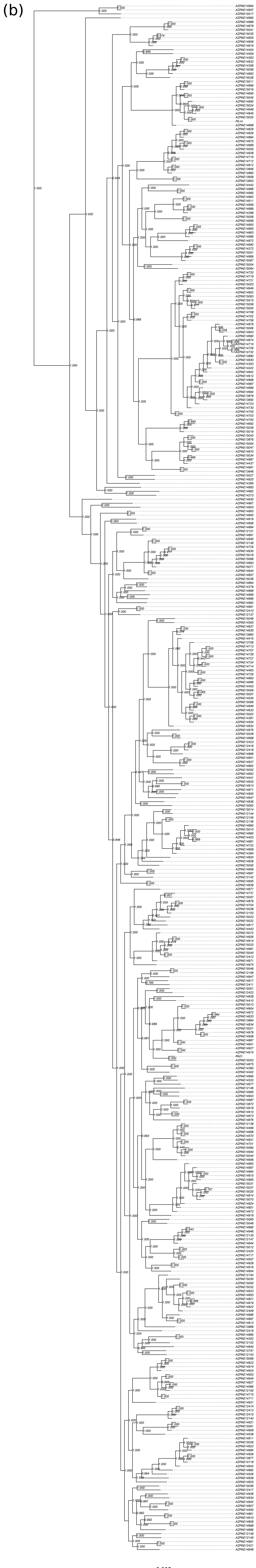

Supplement: Supplementary file 2 [file EVA-13-781-s002.pdf]

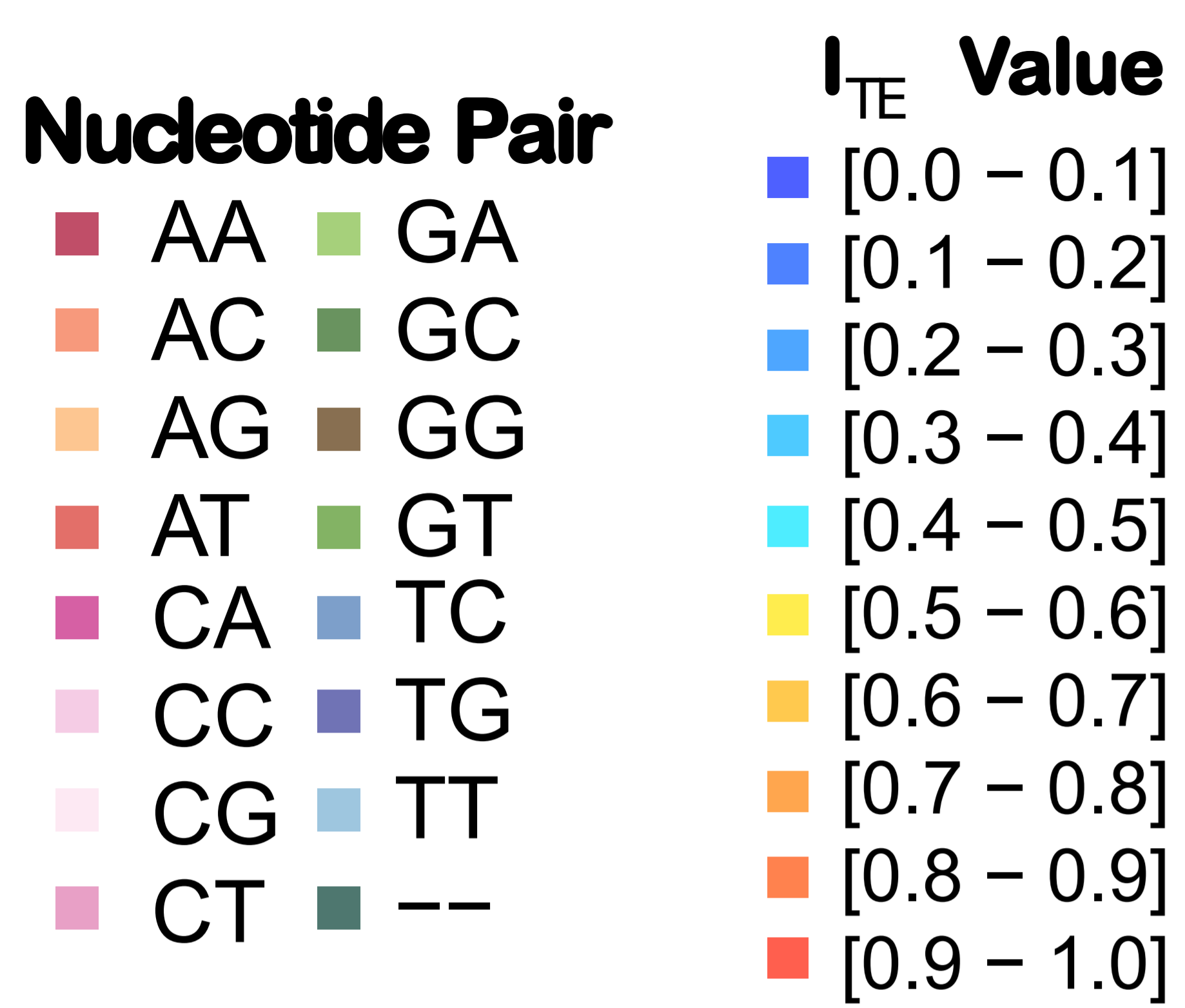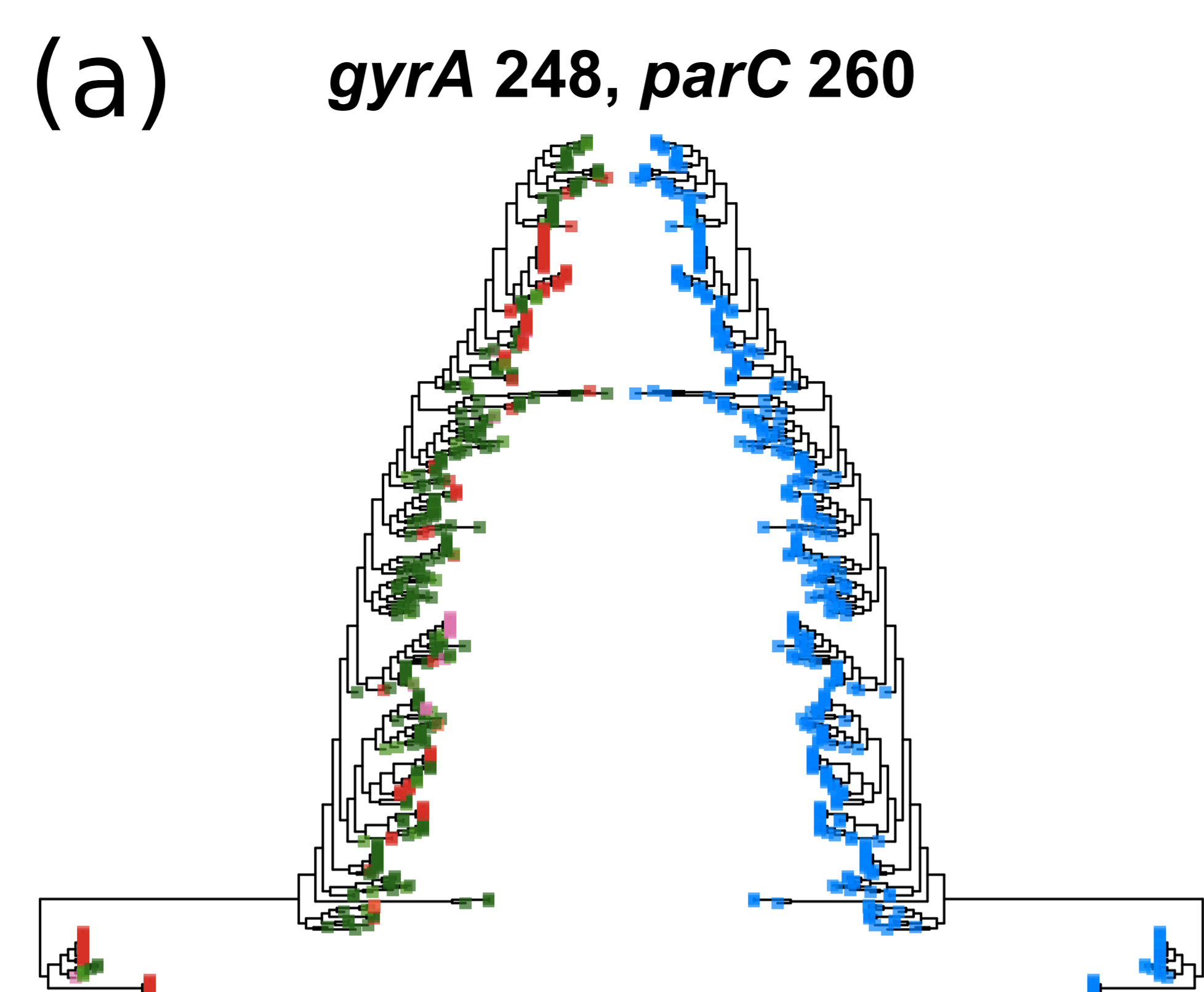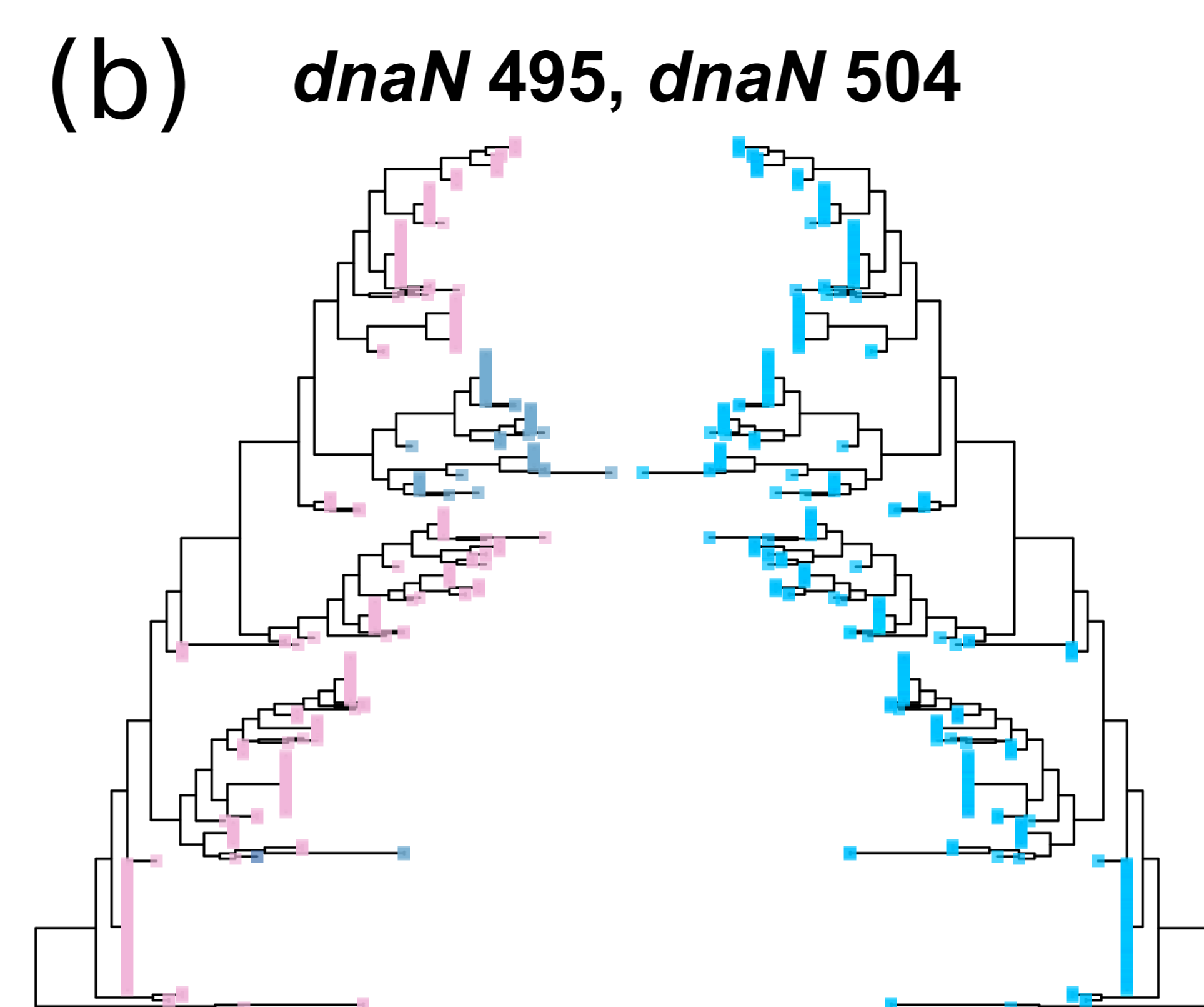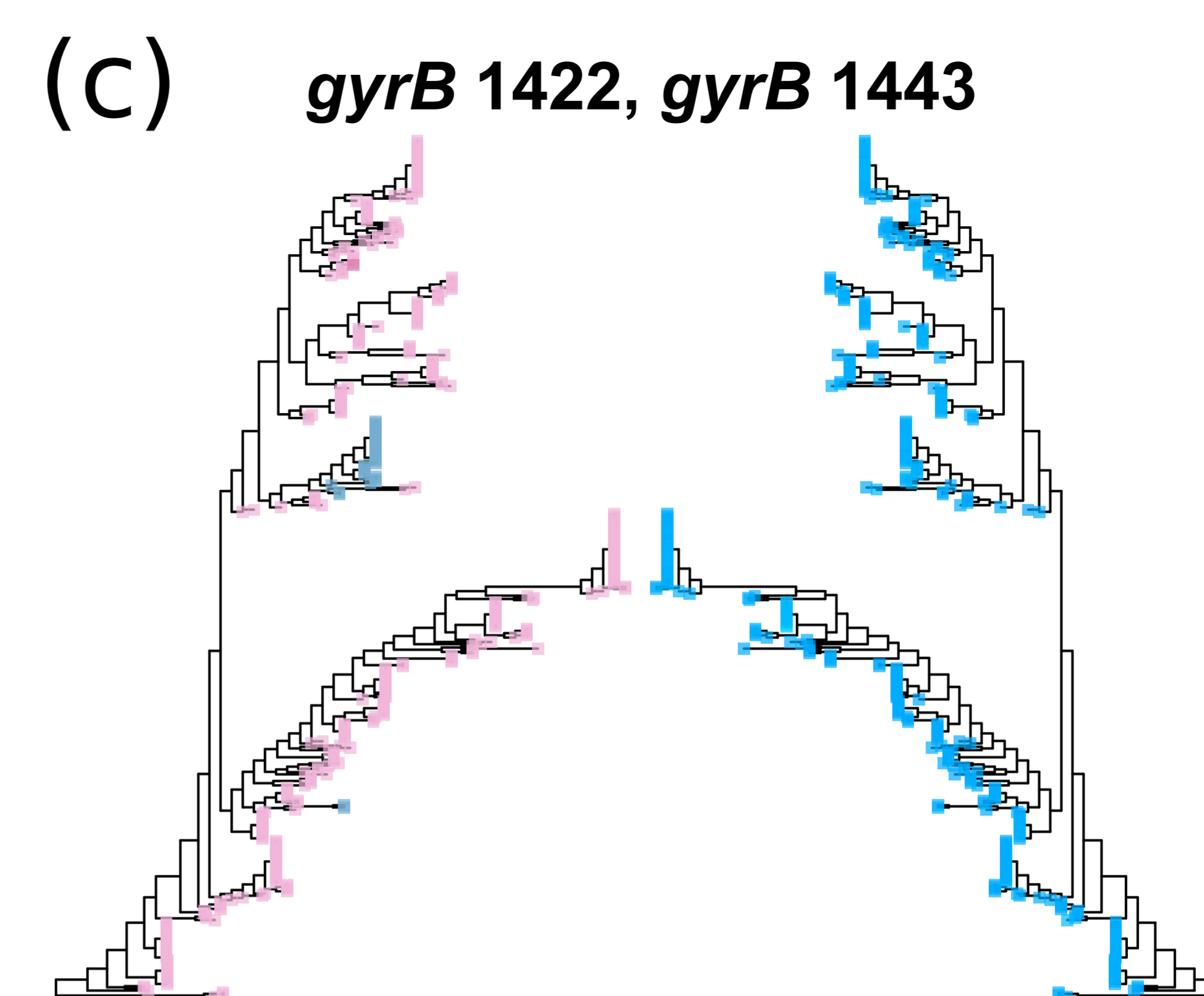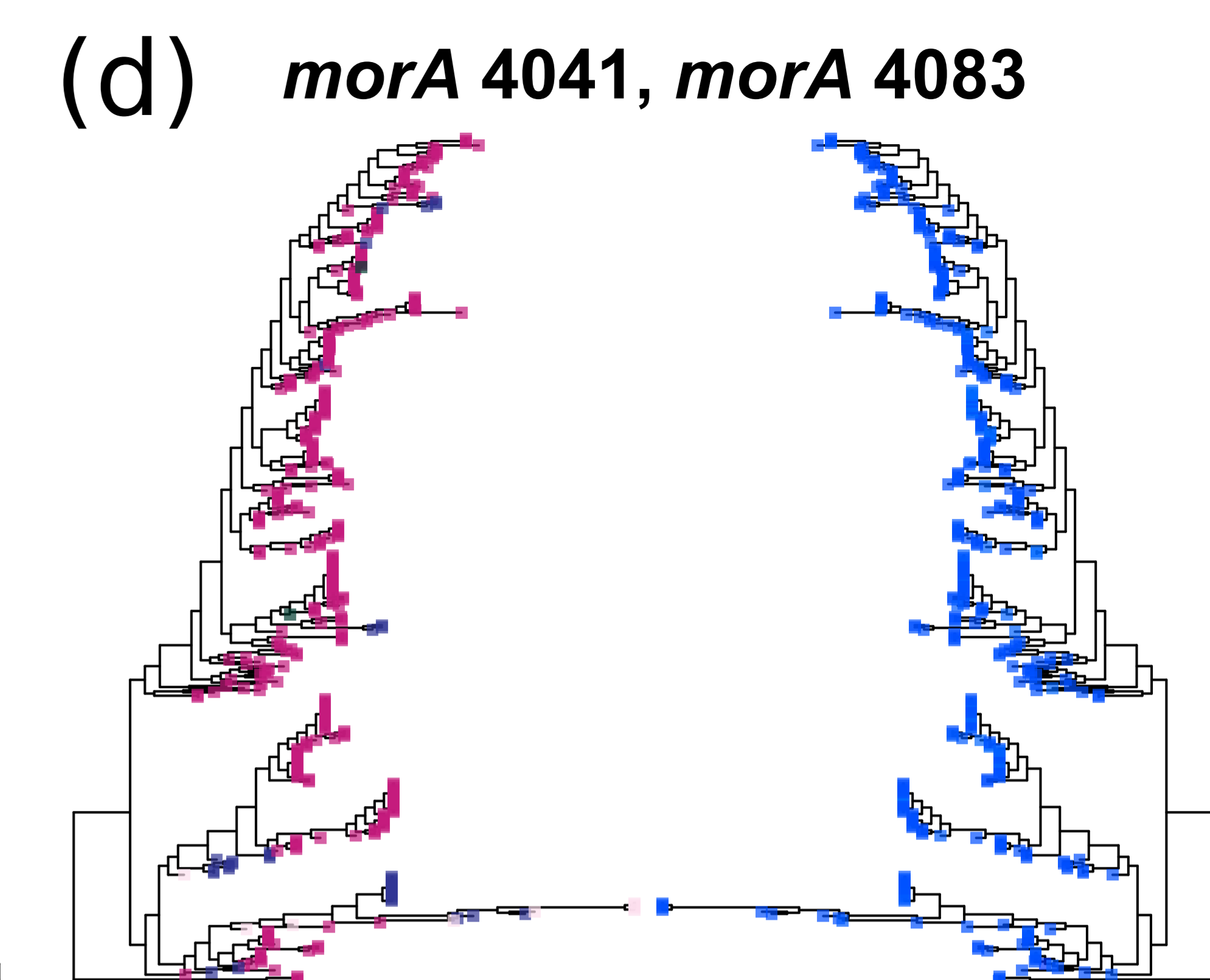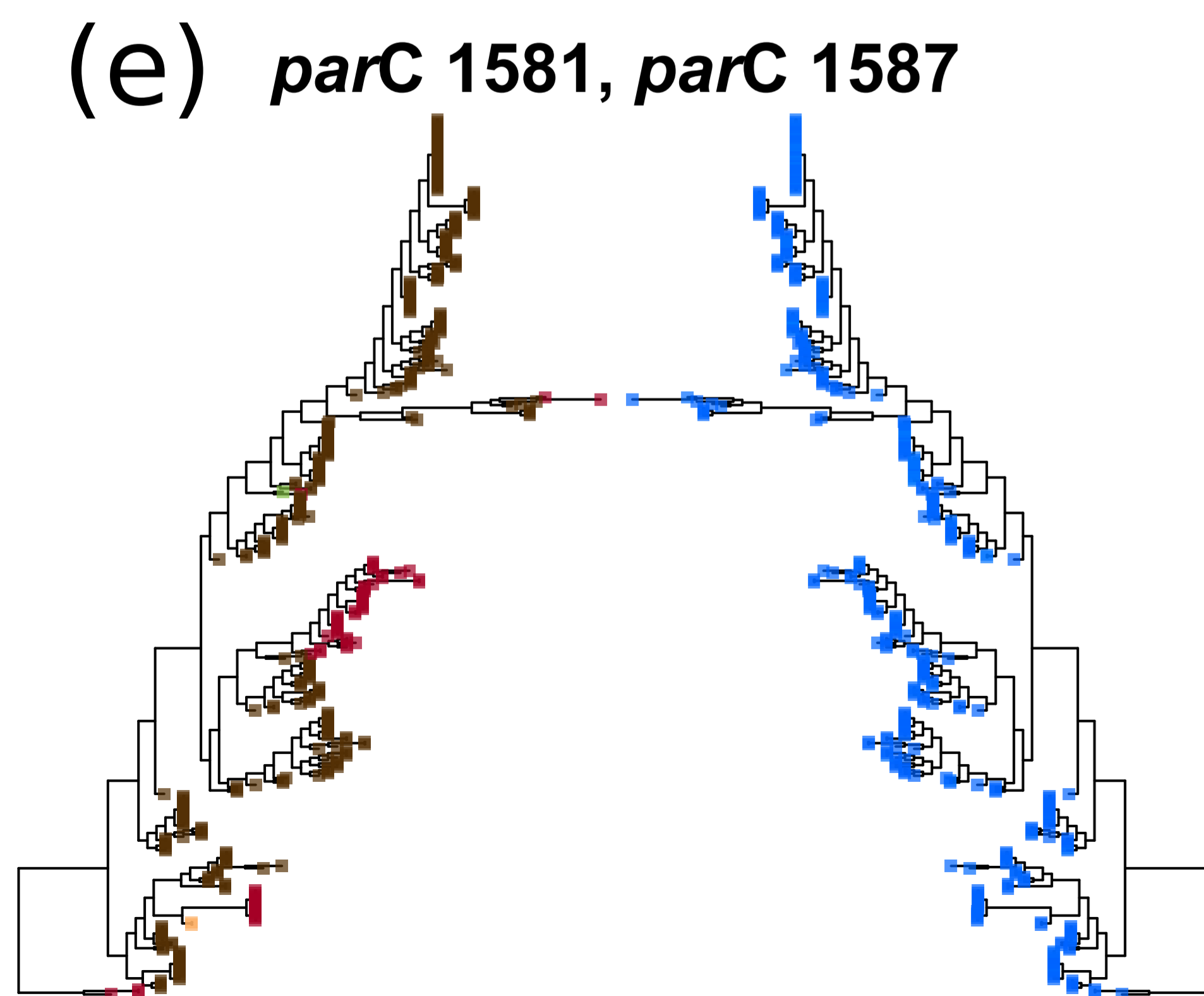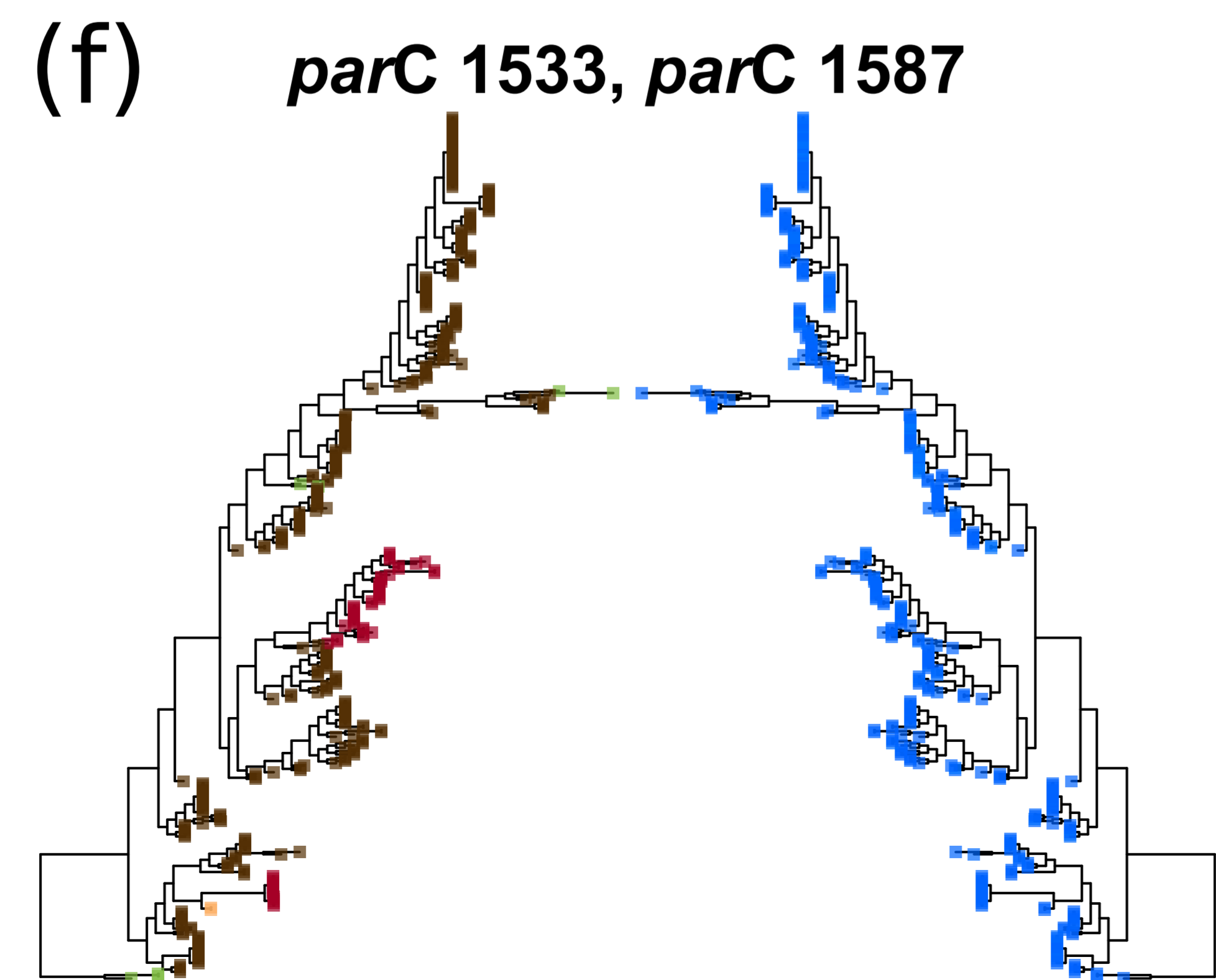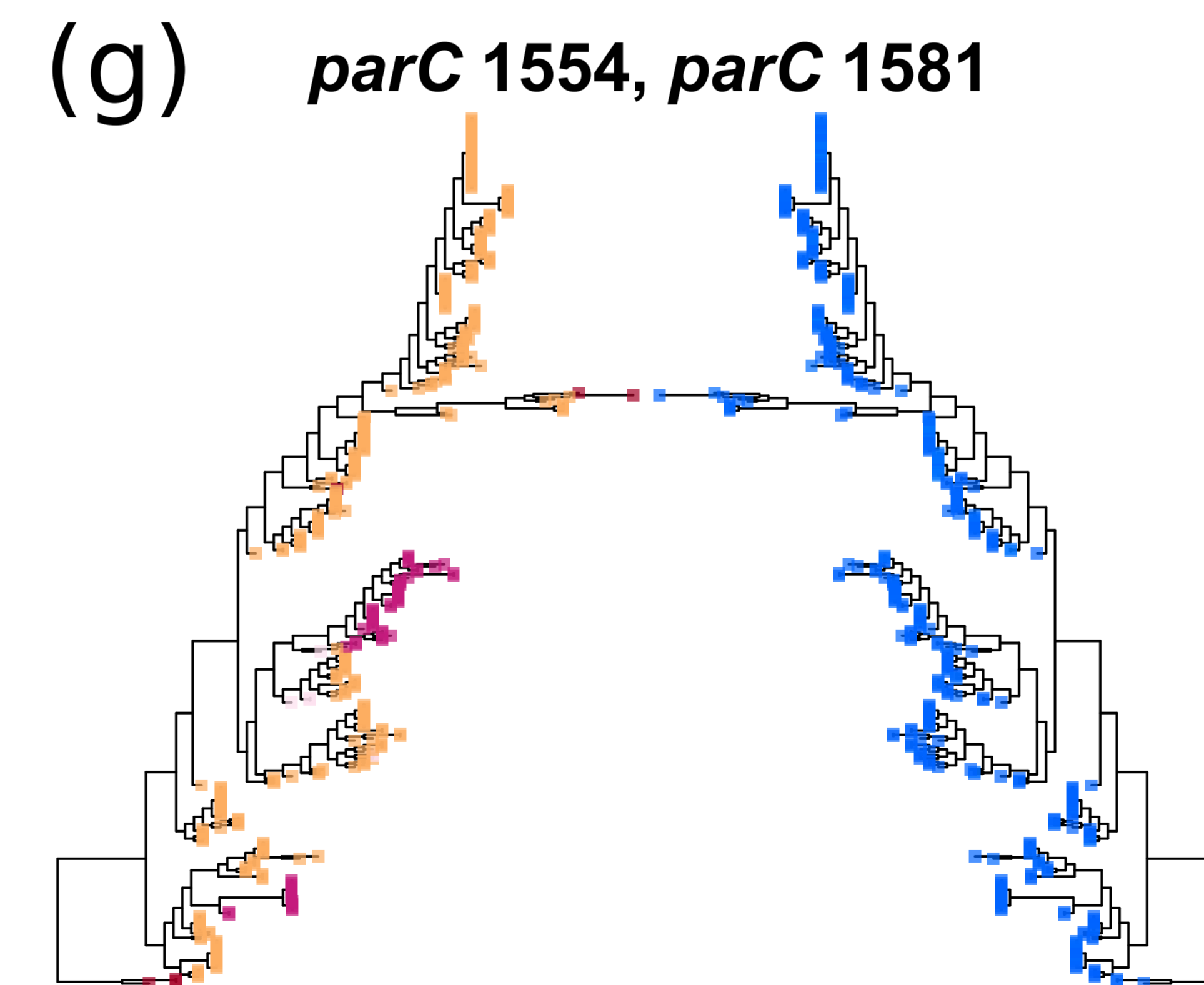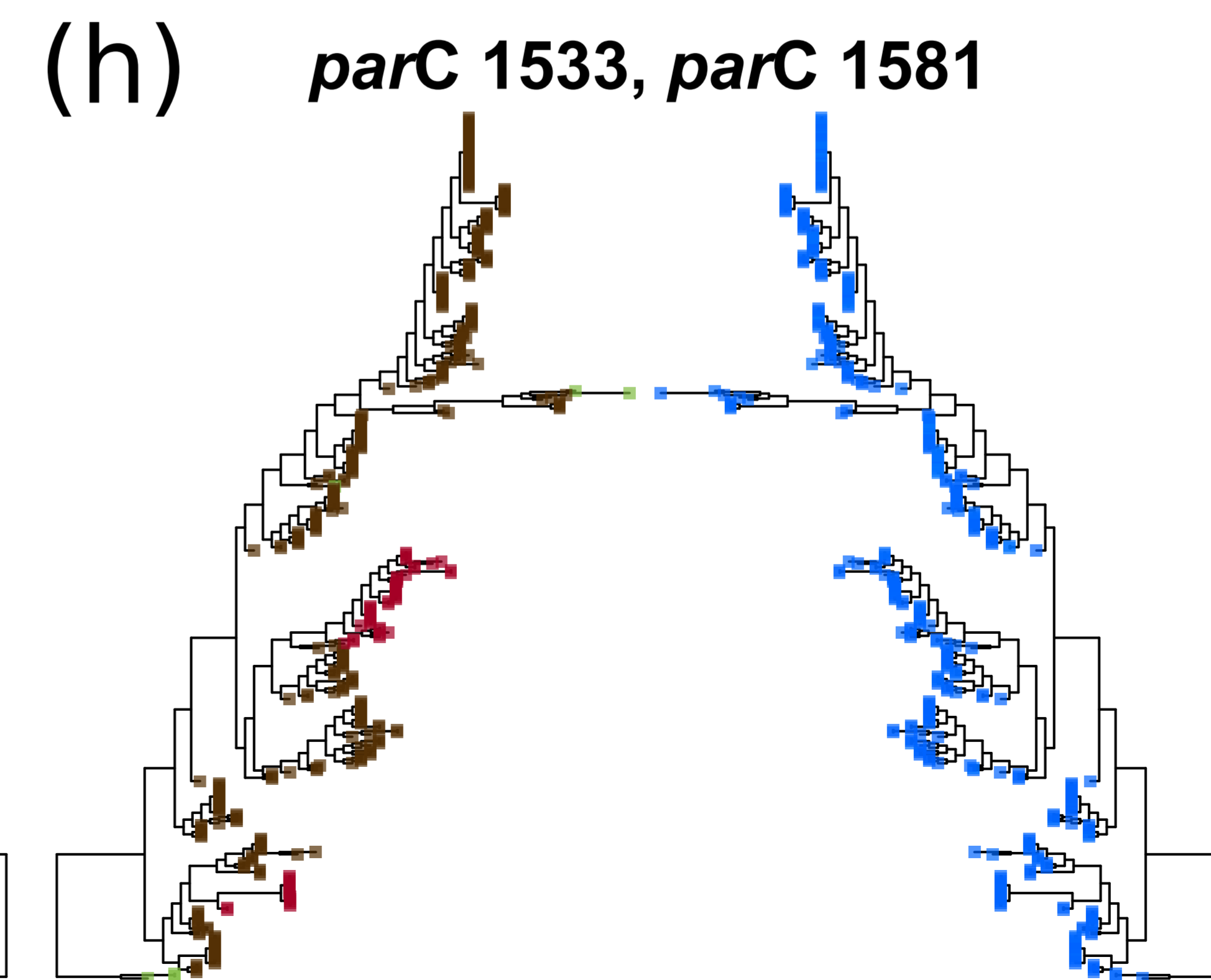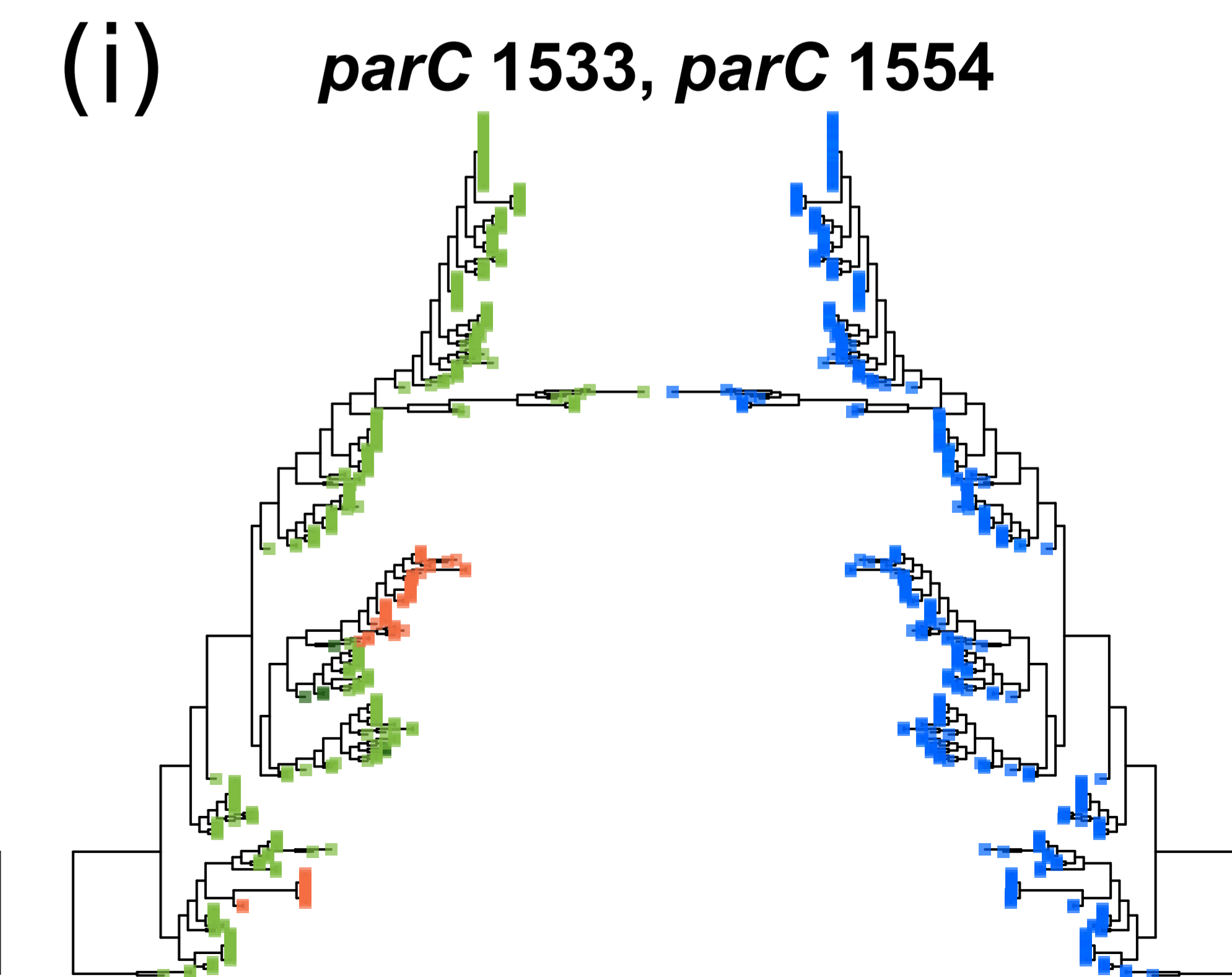

Supplement: Supplementary file 3 [file EVA-13-781-s003.pdf]

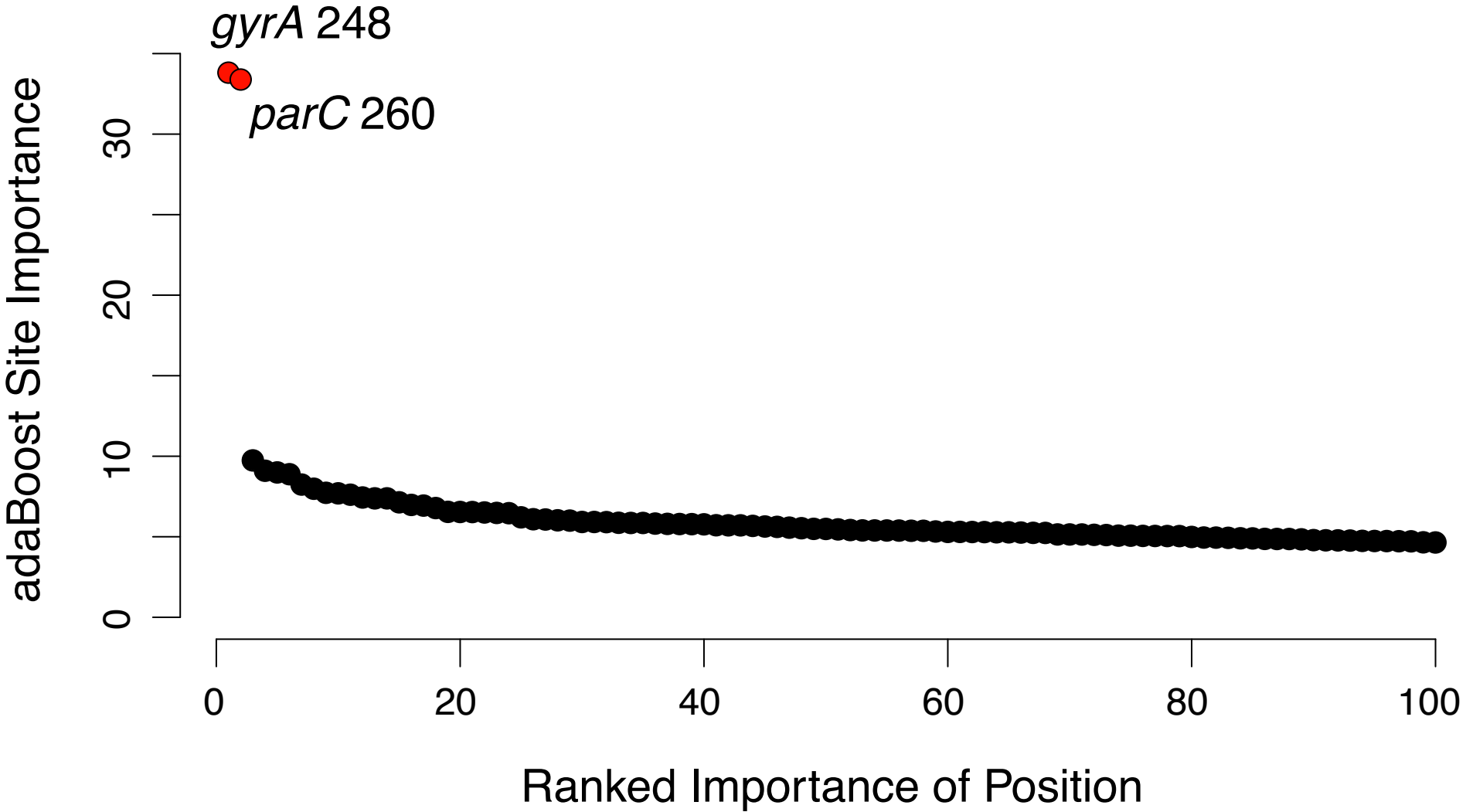

Supplement: Supplementary file 4 [file EVA-13-781-s004.pdf]

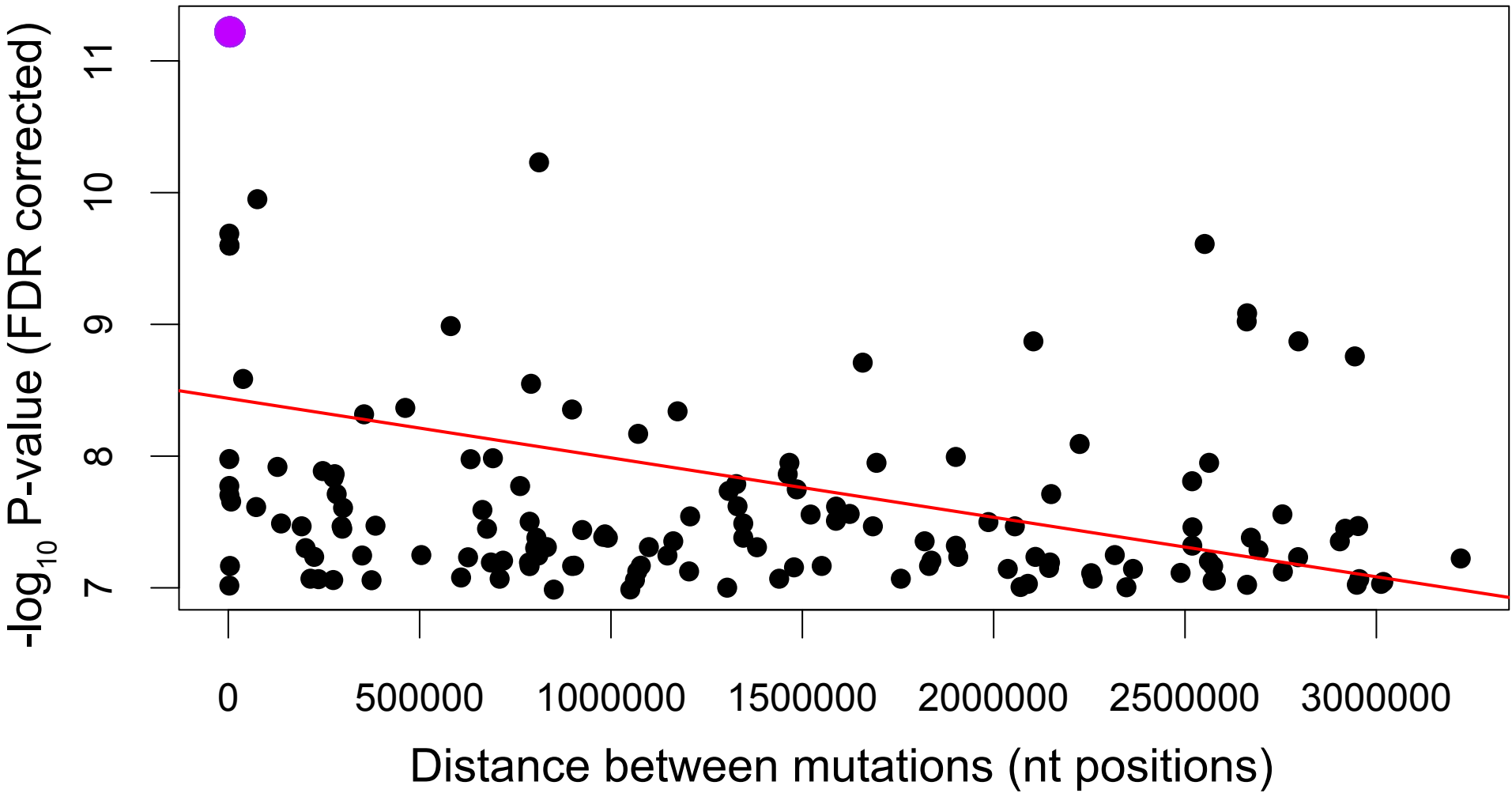

Supplement: Supplementary file 5 [file EVA-13-781-s005.pdf]
