# Supplementary material for: Identifying the drivers of computationally detected correlated evolution among sites under antibiotic selection
Source: Evol Appl. 2020 Feb 13;13(4):781–93. doi: 10.1111/eva.12900 (PMC7086105; doi:10.1111/eva.12900)
Supplement: Supplementary file 6 [file EVA-13-781-s006.pdf]

1 Supplementary Text

1.1 Additional Methods Details

Alignment algorithm: We developed a custom algorithm to reconstruct an genome alignment, made of concatenated genes (ie: an exome alignment), using as reference a PA14 genome database obtained from `www.pseudomonas.com` (Winsor *et al.*, 2016, accessed Dec 4, 2014). From this database, we extracted the nucleotide sequences for the 5,977 genes, and used each of them as a query for BLASTn (Altschul *et al.*, 1990) searches against a local database containing the 390 draft genomes, as well as the three complete genomes of PA01, PA7 and PA14. Results with more than 90% identity were then used to construct scaffolds, which were subsequently extended, for each of the 390 draft genomes, making sure that the same genomic information was not used more than once. The extended scaffold sequences of each gene were aligned with MUSCLE (Edgar, 2004). We discarded genes present in less than 50% of strains with $\geq$ 90% of the length of the PA14 query (non-gap characters). The remaining genes were concatenated into a single exome alignment with *catfasta2phyml.pl* (Nylander, 2015).

Complexity Hypothesis: The species tree for our exome alignment was reconstructed based on highly networked genes that, according to the complexity hypothesis, are unlikely to be horizontally transferred. According to the complexity hypothesis (Jain *et al.*, 1999; Aris-Brosou, 2005), highly networked genes are those whose products are involved in complex multiprotein interactions such as information processing genes (e.g. rRNA genes, tRNA genes, transcription and translation polymerases). Based on the functional annotations of PA14 genes obtained from the Clusters of Orthologous Groups database (Tatusov *et al.*, 1997; Galperin *et al.*, 2015), we extracted 1,290 information genes (those with COG terms A, B, J, K, and L), and concatenated them into a single alignment used to estimate the species tree.

Analysis of correlated evolution: Recoding our concatenated exome into binary states resulted

in 410,665 polymorphic sites. We estimated that a full pairwise comparison of the polymorphic sites would have required more than one year of computing even when parallelized across twelve computational nodes each with four processors. It is for this reason that we performed a 12 gene by exome analysis.

Modified selection counter-selection protocol: Our modified WT allele replacement protocol is based on a selection counter-selection method (Schweizer, 2008), whereby a vector-borne mutant allele recombines with homologous chromosomal sequences before the delivery vector is itself removed from the targeted genome. Mutant alleles of *gyrA* and *parC* were generated from WT chromosomal DNA by amplification of each locus in paired PCR reactions that overlapped at sequences adjacent to the introduced substitution. One of the overlapping primers coded for the substitution of interest through a mismatch at the target site. The paired amplicons were ligated to an allelic replacement vector using Golden Gate assembly (Engler *et al.*, 2008), which permitted scarless ligation of the PCR products (see Table S2 for details). The vector, derived from pAH79 (Melnyk *et al.*, 2017) and modified for Golden Gate cloning, includes the $TetA$ selectable marker and $sacB$ counter-selection gene. After ligation, vectors were transformed into chemically competent *Escherichia coli* (DH5 λpir). The mutant alleles were transferred into *P. aeruginosa* strains as previously described (Melnyk *et al.*, 2017) via tri-parental conjugation, involving a helper *E. coli* that carries pRK2013 (Figurski and Helinski, 1979). Through a round of selection (LB agar with 100μg/mL Nitrofurantoin and 80μg/mL Tetracycline), we isolated colonies whose genome had recombined with the mutant allele. The recombinants were subsequently counter-selected (LB agar with 5% sucrose) for loss of the plasmid sequences. Mutant constructs were first confirmed by sequencing the region targeted for replacement. To identify constructs least likely to contain secondary mutations outside the sequenced region, we performed competitive fitness assays with a minimum of four independent constructs, and accepted constructs with relatively similar fitness measures for downstream analyses.

Calculating competitive fitness: Competitive fitness was calculated as $\omega = (f_{final} - f_{initial})^{(1/generations)}$, where $f_{initial}$ and f_{final} are the initial and final frequency of focal strains, and the number of generations was based on the dilution factor, calculated as $\log_2(100) \sim 6.64$. The evidence for epistasis (ϵ) was calculated with a multiplicative fitness model (Trindade *et al.*, 2009) such that $\epsilon = W_{WT}W_{AB} - W_A W_B$ where W stands for fitness and the subscripts (WT, A, B, AB) represent the wild-type, single and double mutant genotypes. There is evidence for epistasis when the ϵ value is greater than our measurement error estimated via error propagation.

Adaptive boosting algorithm: Adaptive boosting is a supervised machine learning algorithm that uses the weighted sum of many sequentially fitted classifiers, and is considered the "best out-of-the-box" classifier in part because it yields lower classification error rates while being less susceptible to overfitting (James *et al.*, 2013). Nucleotide sites in our exome alignment were quantified for their importance in predicting levofloxacin resistance by computing the number of fitted classification trees which retain a predictor, in this case nucleotide site. Classification was based on the association with the discrete phenotype of sensitivity or resistance to levofloxacin. Phenotype data were obtained from the original study that published the 390 draft genomes used to construct our alignment (Kos *et al.*, 2015).

Calculating ΔG: To estimate biological effects of synonymous mutations, ΔG values were calculated with the mFold server (Zuker, 2003), based on the QuickFold service with default settings (`unafold.rna.albany.edu/?q=DINAMelt/Quickfold`). Similar to previous studies (Takanami and Zubay, 1964), we calculated the free energy of each mRNA transcript by considering 50 nucleotides upstream and downstream of each substitution. Mean and error values of ΔG_{rel} were calculated using weighted values for all the predicted folding structures returned by QuickFold.

Calculating I_{TE}: Also, to estimate biological effects of synonymous mutations, we calculated the index of translation elongation (I_{TE}: Xia, 2014) with default settings in DAMBE ver. 6

(Xia, 2017). These calculations used the codon frequency of genes that are highly expressed in *P. aeruginosa* (Hilterbrand *et al.*, 2012), and lowly expressed genes calculated using all of the other genes in our alignment. Files containing the codon frequency counts for all strains in our alignment are available from `https://github.com/JDench/Pseudomonas_DAMBE_ITE`.

1.2 Supplementary analyses

While a previous study has shown the excellent specificity of AEGIS at identifying correlated pairs of substitutions from simulated evolution (Nshogozabahizi *et al.*, 2017), the $\approx 127,000$ significantly correlated pairs ($P \leq 0.01$) was much higher than expected and we deemed it beneficial to review the results prior to *in vitro* study. As this study is the first to have tried to identify correlated evolution among pairs of sites in the whole genome of *P. aeruginosa* there was no general dataset against which we could compare our results, instead we performed summary analyses to describe trends in the results. Recall that for reasons of computational time, this study analysed the evidence of correlated evolution between the sites in 12 genes and the rest of the genome. If the results of AEGIS were largely false positives, we would have expected the number of polymorphic sites (*i.e.* sites in our alignment with different nucleotide characters across genotypes) in a gene to be positively correlated with the number of correlated pairs involving substitutions in that gene. We found no evidence that the number of polymorphic sites in the 12 genes was correlated to the number of associated significant pairs (Pearson's ρ, $t = 0.5530$, $df = 10$, $P = 0.5924$). Further, we found no evidence that the proportion of polymorphic sites in a gene (i.e., number of sites divided by gene length) differed between the six focal genes ($gyrA$, $gyrB$, $morA$, $nfxB$, $parC$, $parE$), the additional six genes chosen at random ($dnaA$, $dnaN$, $lpd3$, $ribD$, $rpoB$, $serC$), and any of the other genes in our alignment (ANOVA, $F = 0.1227$, $df = 2$, $P = 0.8845$). This led us to conclude that the results of AEGIS reflected some true evolutionary signal.

While we did not have specific information concerning the *in vitro* effects of most substitutions identified in the results, we could define several descriptive traits. For this, we compared trends among our significantly correlated paired sites to what we would expect by chance (*i.e.* from a null model). If chance alone drove our detection of correlated site pairs, we would expect to detect a paired traits according to their frequency in our exome alignment. We found that the frequency of observed paired traits differs from our null expectation (Fig. S1). As a first assessment we leveraged our *a priori* assumption that mutations in the six focal genes were more likely to show an adaptive response to fluoroquinolone selection compared to the other six genes. If our assumption was correct and the results of AEGIS reflect correlated evolution in response to selection, we would expect more pairs to include mutations in the six focal genes. When comparing the number of correlated pairs which include zero, one, or two sites in the six focal genes, we found that pairs with at least one site in an expected gene are consistently higher than by chance (compare panels a,b in Fig. S1). We next wanted to assess if correlated pairs of potentially adaptive substitutions (i.e. nonsynonymous) were detected more often than by chance. While synonymous substitutions may be selected for (Agashe *et al.*, 2016; Bailey *et al.*, 2014), in response to antibiotic selection we assumed that only nonsynonymous substitutions were likely to be adaptive. We looked for differences in the expected and observed number of correlated pairs where zero, one or two sites were nonsynonymous (compare Fig. S1 c,d). We found that nonsynonymous pairs are dramatically underrepresented comprising only 2 pairs with at least medium (*parC* 786 and PA14_34000 967 - hypothetical type VI secretion protein - $10^{-7} \leq P \leq 10^{-6}$) and strong (*gyrA* 248 and *parC* 260, $P \leq 10^{-11}$) evidence for correlated evolution respectively. We interpret the higher than expected number of synonymous pairs to suggest that hitchhiking (Maynard Smith and Haigh, 1974), possibly as “cohorts” (Lang *et al.*, 2013), explains the majority of correlated pairs identified by AEGIS.

References

Agashe, D., Sane, M., Phalnikar, K., Diwan, G. D., Habibullah, A., Martinez-Gomez, N. C., Sahasrabuddhe, V., Polachek, W., Wang, J., Chubiz, L. M., and Marx, C. J. 2016. Large-effect beneficial synonymous mutations mediate rapid and parallel adaptation in a bacterium. *Molecular Biology and Evolution*, 33(6): 1542–1553.

Alfaro, E., Gámez, M., and García, N. 2013. adabag: An R package for classification with boosting and bagging. *Journal of Statistical Software*, 54(2): 1–35.

Altschul, S. F., Gish, W., Miller, W., Myers, E. W., and Lipman, D. J. 1990. Basic local alignment search tool. *Journal of Molecular Biology*, 215(3): 403 – 410.

Aris-Brosou, S. 2005. Determinants of adaptive evolution at the molecular level: the extended complexity hypothesis. *Mol Biol Evol*, 22(2): 200–9.

Bailey, S. F., Hinz, A., and Kassen, R. 2014. Adaptive synonymous mutations in an experimentally evolved *Pseudomonas fluorescens* population. *Nature Communications*, 5: 4076–4076.

Edgar, R. C. 2004. MUSCLE: multiple sequence alignment with high accuracy and high throughput. *Nucleic Acids Research*, 32(5): 1792–1797.

Engler, C., Kandzia, R., and Marillonnet, S. 2008. A one pot, one step, precision cloning method with high throughput capability. *PLoS ONE*, 3(11): 1–7.

Figurski, D. H. and Helinski, D. R. 1979. Replication of an origin-containing derivative of plasmid RK2 dependent on a plasmid function provided in trans. *Proceedings of the National Academy of Sciences of the United States of America*, 76(4): 1648–1652.

Galperin, M. Y., Makarova, K. S., Wolf, Y. I., and Koonin, E. V. 2015. Expanded microbial genome coverage and improved protein family annotation in the COG database. *Nucleic Acids Research*, 43(D1): D261–D269.

Hilterbrand, A., Saelens, J., and Putonti, C. 2012. CBDB: The codon bias database. *BMC Bioinformatics*, 13(1): 62.

Holloway, B. 1955. Genetic recombination in pseudomonas-aeruginosa. *Journal of general microbiology*, 13(3): 572–581.

Jain, R., Rivera, M. C., and Lake, J. A. 1999. Horizontal gene transfer among genomes: The complexity hypothesis. *Proceedings of the National Academy of Sciences of the United States of America*, 96(7): 3801–3806.

James, G., Witten, D., Hastie, T., and Tibshirani, R. 2013. *An introduction to statistical machine learning with applications in R*. Springer.

Kos, V. N., Déraspe, M., McLaughlin, R. E., Whiteaker, J. D., Roy, P. H., Alm, R. A., Corbeil, J., and Gardner, H. 2015. The resistome of *Pseudomonas aeruginosa* in relationship to phenotypic susceptibility. *Antimicrobial Agents and Chemotherapy*, 59(1): 427–436.

Lang, G. I., Rice, D. P., Hickman, M. J., Sodergren, E., Weinstock, G. M., Botstein, D., and Desai, M. M. 2013. Pervasive genetic hitchhiking and clonal interference in forty evolving yeast populations. *Nature*, 500(7464): 571–4.

Maynard Smith, J. and Haigh, J. 1974. The hitch-hiking effect of a favourable gene. *Genetical Research*, 23(01): 23–35.

Melnyk, A. H., McCloskey, N., Hinz, A. J., Dettman, J., and Kassen, R. 2017. Evolution of cost-free resistance under fluctuating drug selection in *Pseudomonas aeruginosa*. *mSphere*, 2(4).

Nshogozabahizi, J. C., Dench, J., and Aris-Brosou, S. 2017. Widespread historical contingency in influenza viruses. *Genetics*, 205(1): 409–420.

Nylander, J. 2015. catfasta2phyml. `https://github.com/nylander/catfasta2phyml`.

Price, M. N., Dehal, P. S., and Arkin, A. P. 2010. FastTree 2 - approximately maximum-likelihood trees for large alignments. *PLoS ONE*, 5(3).

Rahme, L. G., Stevens, E. J., Wolfort, S. F., Shao, J., Tompkins, R. G., and Ausubel, F. M. 1995. Common virulence factors for bacterial pathogenicity in plants and animals. *Science*, 268(5219): 1899–1902.

Roy, P. H., Tetu, S. G., Larouche, A., Elbourne, L., Tremblay, S., Ren, Q., Dodson, R., Harkins, D., Shay, R., Watkins, K., Mahamoud, Y., and Paulsen, I. T. 2010. Complete genome sequence of the multiresistant taxonomic outlier *Pseudomonas aeruginosa* PA7. *PLoS ONE*, 5(1): e8842.

Schweizer, H. P. 2008. Bacterial genetics: past achievements, present state of the field, and future challenges. *BioTechniques*, 44: 633–641.

Takanami, M. and Zubay, G. 1964. An estimate of the size of the ribosomal site for messenger RNA binding. *Proceedings of the National Academy of Sciences of the United States of America*, 51(5): 834–839.

Tatusov, R. L., Koonin, E. V., and Lipman, D. J. 1997. A genomic perspective on protein families. *Science*, 278(5338): 631–637.

Trindade, S., Sousa, A., Xavier, K. B., Dionisio, F., Ferreira, M. G., and Gordo, I. 2009. Positive epistasis drives the acquisition of multidrug resistance. *PLoS Genetics*, 5(7): e1000578–e1000578.

Winsor, G. L., Griffiths, E. J., Lo, R., Dhillon, B. K., Shay, J. A., and Brinkman, F. S. L. 2016. Enhanced annotations and features for comparing thousands of pseudomonas genomes in the pseudomonas genome database. *Nucleic Acids Res*, 44(D1): D646–53.

Xia, X. 2014. A major controversy in codon-anticodon adaptation resolved by a new codon usage index. *Genetics*, 199(2): 573–579.

Xia, X. 2017. DAMBE6: New tools for microbial genomics, phylogenetics, and molecular evolution. *Journal of Heredity*, 108(4): 431–437.

Zuker, M. 2003. Mfold web server for nucleic acid folding and hybridization prediction. *Nucleic Acids Research*, 31(13): 3406–3415.

SI Tables

Table S1. Table of strains included in whole exome alignment. Where known we provide the name of isolates used in our study along with the year, country and origin as per the cited reference paper.

Isolate	Year of isolation	Country	City	Reference
AZPAE12135	2005	United States	New York	(Kos *et al.*, 2015)
AZPAE12136	2005	United States	New York	(Kos *et al.*, 2015)
AZPAE12137	2005	United States	New York	(Kos *et al.*, 2015)
AZPAE12138	2005	United States	New York	(Kos *et al.*, 2015)
AZPAE12140	2005	United States	New York	(Kos *et al.*, 2015)
AZPAE12142	2005	United States	New York	(Kos *et al.*, 2015)
AZPAE12143	2005	United States	New York	(Kos *et al.*, 2015)
AZPAE12144	2005	United States	New York	(Kos *et al.*, 2015)
AZPAE12145	2005	United States	New York	(Kos *et al.*, 2015)
AZPAE12146	2005	United States	New York	(Kos *et al.*, 2015)
AZPAE12147	2005	United States	New York	(Kos *et al.*, 2015)
AZPAE12148	2005	United States	New York	(Kos *et al.*, 2015)
AZPAE12149	2005	United States	New York	(Kos *et al.*, 2015)
AZPAE12150	2005	United States	New York	(Kos *et al.*, 2015)
AZPAE12151	2005	United States	New York	(Kos *et al.*, 2015)
AZPAE12152	2005	United States	New York	(Kos *et al.*, 2015)
AZPAE12153	2005	United States	New York	(Kos *et al.*, 2015)
AZPAE12154	2005	United States	New York	(Kos *et al.*, 2015)
AZPAE12155	2005	United States	New York	(Kos *et al.*, 2015)
AZPAE12156	2005	United States	New York	(Kos *et al.*, 2015)
AZPAE12409	2007	United States	Cleveland	(Kos *et al.*, 2015)
AZPAE12410	2007	United States	Cleveland	(Kos *et al.*, 2015)
AZPAE12411	2007	United States	Cleveland	(Kos *et al.*, 2015)
AZPAE12412	2007	United States	Cleveland	(Kos *et al.*, 2015)
AZPAE12413	2007	United States	Cleveland	(Kos *et al.*, 2015)
AZPAE12414	2007	United States	Cleveland	(Kos *et al.*, 2015)
AZPAE12415	2007	United States	Cleveland	(Kos *et al.*, 2015)
AZPAE12416	2007	United States	Cleveland	(Kos *et al.*, 2015)
AZPAE12417	2007	United States	Cleveland	(Kos *et al.*, 2015)
AZPAE12418	2007	United States	Cleveland	(Kos *et al.*, 2015)
AZPAE12419	2007	United States	Cleveland	(Kos *et al.*, 2015)
AZPAE12420	2007	United States	Cleveland	(Kos *et al.*, 2015)
AZPAE12421	2007	United States	Cleveland	(Kos *et al.*, 2015)

Continued on next page

Table S1 – continued from previous page

Isolat	Year of isolation	Country	City	Reference
AZPAE12422	2007	United States	Cleveland	(Kos *et al.*, 2015)
AZPAE12423	2007	United States	Cleveland	(Kos *et al.*, 2015)
AZPAE13756	2009	Canada	unknown	(Kos *et al.*, 2015)
AZPAE13757	2009	Canada	unknown	(Kos *et al.*, 2015)
AZPAE13848	2010	India	unknown	(Kos *et al.*, 2015)
AZPAE13850	2010	India	unknown	(Kos *et al.*, 2015)
AZPAE13853	2010	India	unknown	(Kos *et al.*, 2015)
AZPAE13856	2010	India	unknown	(Kos *et al.*, 2015)
AZPAE13858	2010	India	unknown	(Kos *et al.*, 2015)
AZPAE13860	2010	India	unknown	(Kos *et al.*, 2015)
AZPAE13864	2010	India	unknown	(Kos *et al.*, 2015)
AZPAE13866	2010	China	unknown	(Kos *et al.*, 2015)
AZPAE13872	2010	Mexico	unknown	(Kos *et al.*, 2015)
AZPAE13876	2010	Portugal	unknown	(Kos *et al.*, 2015)
AZPAE13877	2010	Romania	unknown	(Kos *et al.*, 2015)
AZPAE13879	2010	Argentina	unknown	(Kos *et al.*, 2015)
AZPAE13880	2010	Mexico	unknown	(Kos *et al.*, 2015)
AZPAE14352	2010	France	unknown	(Kos *et al.*, 2015)
AZPAE14353	2010	France	unknown	(Kos *et al.*, 2015)
AZPAE14359	2010	China	Shatin	(Kos *et al.*, 2015)
AZPAE14372	2010	China	Hong Kong	(Kos *et al.*, 2015)
AZPAE14373	2010	Germany	München	(Kos *et al.*, 2015)
AZPAE14379	2010	Germany	Heidelberg	(Kos *et al.*, 2015)
AZPAE14381	2010	Spain	Bilbao	(Kos *et al.*, 2015)
AZPAE14390	2011	China	Hong Kong	(Kos *et al.*, 2015)
AZPAE14393	2011	Spain	Madrid	(Kos *et al.*, 2015)
AZPAE14394	2011	Spain	Madrid	(Kos *et al.*, 2015)
AZPAE14395	2011	Spain	Bilbao	(Kos *et al.*, 2015)
AZPAE14398	2011	Germany	München	(Kos *et al.*, 2015)
AZPAE14402	2011	France	Rouen	(Kos *et al.*, 2015)
AZPAE14403	2011	France	Rouen	(Kos *et al.*, 2015)
AZPAE14404	2012	China	Shatin	(Kos *et al.*, 2015)
AZPAE14410	2012	Germany	Heidelberg	(Kos *et al.*, 2015)
AZPAE14415	2009	Portugal	unknown	(Kos *et al.*, 2015)
AZPAE14422	2009	United States	Roseburg	(Kos *et al.*, 2015)
AZPAE14437	2010	Canada	unknown	(Kos *et al.*, 2015)
AZPAE14441	2010	Taiwan	unknown	(Kos *et al.*, 2015)
AZPAE14442	2010	Taiwan	unknown	(Kos *et al.*, 2015)

Continued on next page

Table S1 – continued from previous page

Isolat	Year of isolation	Country	City	Reference
AZPAE14443	2010	United States	unknown	(Kos *et al.*, 2015)
AZPAE14453	2011	United States	Detroit	(Kos *et al.*, 2015)
AZPAE14463	2011	Colombia	Bogota	(Kos *et al.*, 2015)
AZPAE14499	2011	Spain	Santander	(Kos *et al.*, 2015)
AZPAE14505	2011	France	Paris	(Kos *et al.*, 2015)
AZPAE14509	2011	France	Nantes	(Kos *et al.*, 2015)
AZPAE14526	2010	Spain	Palma de Mallorca	(Kos *et al.*, 2015)
AZPAE14533	2011	Germany	Koln	(Kos *et al.*, 2015)
AZPAE14535	2010	Spain	Santander	(Kos *et al.*, 2015)
AZPAE14538	2010	China	Shatin	(Kos *et al.*, 2015)
AZPAE14550	2010	China	Beijing	(Kos *et al.*, 2015)
AZPAE14554	2010	Spain	Santander	(Kos *et al.*, 2015)
AZPAE14557	2010	Germany	Koln	(Kos *et al.*, 2015)
AZPAE14566	2011	China	Shatin	(Kos *et al.*, 2015)
AZPAE14570	2010	Germany	unknown	(Kos *et al.*, 2015)
AZPAE14687	2012	Mexico	unknown	(Kos *et al.*, 2015)
AZPAE14688	2012	Mexico	unknown	(Kos *et al.*, 2015)
AZPAE14689	2012	Mexico	unknown	(Kos *et al.*, 2015)
AZPAE14690	2012	Romania	unknown	(Kos *et al.*, 2015)
AZPAE14691	2012	United States	unknown	(Kos *et al.*, 2015)
AZPAE14692	2012	United States	unknown	(Kos *et al.*, 2015)
AZPAE14693	2012	Romania	unknown	(Kos *et al.*, 2015)
AZPAE14694	2012	Romania	unknown	(Kos *et al.*, 2015)
AZPAE14695	2012	Israel	unknown	(Kos *et al.*, 2015)
AZPAE14697	2012	Israel	unknown	(Kos *et al.*, 2015)
AZPAE14698	2012	Israel	unknown	(Kos *et al.*, 2015)
AZPAE14699	2012	United States	unknown	(Kos *et al.*, 2015)
AZPAE14700	2012	Philippines	unknown	(Kos *et al.*, 2015)
AZPAE14701	2012	Philippines	unknown	(Kos *et al.*, 2015)
AZPAE14702	2012	Philippines	unknown	(Kos *et al.*, 2015)
AZPAE14703	2012	Philippines	unknown	(Kos *et al.*, 2015)
AZPAE14704	2012	Greece	unknown	(Kos *et al.*, 2015)
AZPAE14705	2012	Greece	unknown	(Kos *et al.*, 2015)
AZPAE14706	2012	Greece	unknown	(Kos *et al.*, 2015)
AZPAE14707	2012	Greece	unknown	(Kos *et al.*, 2015)
AZPAE14708	2012	Greece	unknown	(Kos *et al.*, 2015)
AZPAE14710	2012	United States	unknown	(Kos *et al.*, 2015)
AZPAE14711	2012	Venezuela	unknown	(Kos *et al.*, 2015)

Continued on next page

Table S1 – continued from previous page

Isolat	Year of isolation	Country	City	Reference
AZPAE14712	2012	Venezuela	unknown	(Kos *et al.*, 2015)
AZPAE14713	2012	Venezuela	unknown	(Kos *et al.*, 2015)
AZPAE14714	2012	Venezuela	unknown	(Kos *et al.*, 2015)
AZPAE14715	2012	Venezuela	unknown	(Kos *et al.*, 2015)
AZPAE14716	2012	Venezuela	unknown	(Kos *et al.*, 2015)
AZPAE14717	2012	United States	unknown	(Kos *et al.*, 2015)
AZPAE14718	2012	United States	unknown	(Kos *et al.*, 2015)
AZPAE14719	2012	Colombia	unknown	(Kos *et al.*, 2015)
AZPAE14720	2012	Colombia	unknown	(Kos *et al.*, 2015)
AZPAE14721	2012	Colombia	unknown	(Kos *et al.*, 2015)
AZPAE14722	2012	Italy	unknown	(Kos *et al.*, 2015)
AZPAE14723	2012	Italy	unknown	(Kos *et al.*, 2015)
AZPAE14724	2012	Italy	unknown	(Kos *et al.*, 2015)
AZPAE14725	2012	United States	unknown	(Kos *et al.*, 2015)
AZPAE14726	2012	United States	unknown	(Kos *et al.*, 2015)
AZPAE14727	2012	United States	unknown	(Kos *et al.*, 2015)
AZPAE14728	2012	United States	unknown	(Kos *et al.*, 2015)
AZPAE14729	2012	Italy	unknown	(Kos *et al.*, 2015)
AZPAE14730	2012	Italy	unknown	(Kos *et al.*, 2015)
AZPAE14731	2012	Italy	unknown	(Kos *et al.*, 2015)
AZPAE14732	2012	United States	unknown	(Kos *et al.*, 2015)
AZPAE14809	2004	India	Mumbai	(Kos *et al.*, 2015)
AZPAE14810	2004	India	Mumbai	(Kos *et al.*, 2015)
AZPAE14811	2004	India	Mumbai	(Kos *et al.*, 2015)
AZPAE14812	2004	India	Mumbai	(Kos *et al.*, 2015)
AZPAE14813	2004	India	Mumbai	(Kos *et al.*, 2015)
AZPAE14814	2004	France	Besancon	(Kos *et al.*, 2015)
AZPAE14815	2004	France	Besancon	(Kos *et al.*, 2015)
AZPAE14816	2004	France	Besancon	(Kos *et al.*, 2015)
AZPAE14817	2004	France	Besancon	(Kos *et al.*, 2015)
AZPAE14818	2004	France	Besancon	(Kos *et al.*, 2015)
AZPAE14819	2004	Brazil	Sao Paulo	(Kos *et al.*, 2015)
AZPAE14820	2004	Brazil	Sao Paulo	(Kos *et al.*, 2015)
AZPAE14821	2004	Brazil	Sao Paulo	(Kos *et al.*, 2015)
AZPAE14822	2004	Brazil	Sao Paulo	(Kos *et al.*, 2015)
AZPAE14823	2005	Germany	Koln	(Kos *et al.*, 2015)
AZPAE14824	2005	Germany	Koln	(Kos *et al.*, 2015)
AZPAE14825	2005	Germany	Koln	(Kos *et al.*, 2015)

Continued on next page

Table S1 – continued from previous page

Isolat	Year of isolation	Country	City	Reference
AZPAE14826	2005	United States	Detroit	(Kos *et al.*, 2015)
AZPAE14827	2005	United States	Detroit	(Kos *et al.*, 2015)
AZPAE14828	2005	United States	Detroit	(Kos *et al.*, 2015)
AZPAE14829	2005	United States	Detroit	(Kos *et al.*, 2015)
AZPAE14830	2005	Argentina	Victoria	(Kos *et al.*, 2015)
AZPAE14831	2005	Argentina	Victoria	(Kos *et al.*, 2015)
AZPAE14832	2005	Argentina	Victoria	(Kos *et al.*, 2015)
AZPAE14833	2005	Argentina	Victoria	(Kos *et al.*, 2015)
AZPAE14834	2005	Argentina	Victoria	(Kos *et al.*, 2015)
AZPAE14835	2006	China	Beijing	(Kos *et al.*, 2015)
AZPAE14836	2006	China	Beijing	(Kos *et al.*, 2015)
AZPAE14837	2006	China	Beijing	(Kos *et al.*, 2015)
AZPAE14838	2006	China	Beijing	(Kos *et al.*, 2015)
AZPAE14839	2006	China	Beijing	(Kos *et al.*, 2015)
AZPAE14840	2006	China	Beijing	(Kos *et al.*, 2015)
AZPAE14841	2006	China	Beijing	(Kos *et al.*, 2015)
AZPAE14842	2006	United States	Roseburg	(Kos *et al.*, 2015)
AZPAE14843	2006	United States	Roseburg	(Kos *et al.*, 2015)
AZPAE14844	2006	United States	Roseburg	(Kos *et al.*, 2015)
AZPAE14845	2006	Germany	Koln	(Kos *et al.*, 2015)
AZPAE14846	2006	France	Nantes	(Kos *et al.*, 2015)
AZPAE14847	2006	France	Nantes	(Kos *et al.*, 2015)
AZPAE14848	2006	France	Nantes	(Kos *et al.*, 2015)
AZPAE14850	2006	France	Nantes	(Kos *et al.*, 2015)
AZPAE14851	2006	France	Nantes	(Kos *et al.*, 2015)
AZPAE14852	2005	Brazil	Sao Paulo	(Kos *et al.*, 2015)
AZPAE14853	2007	Brazil	Curitiba	(Kos *et al.*, 2015)
AZPAE14855	2007	France	Paris	(Kos *et al.*, 2015)
AZPAE14856	2007	France	Paris	(Kos *et al.*, 2015)
AZPAE14857	2007	France	Paris	(Kos *et al.*, 2015)
AZPAE14858	2007	France	Paris	(Kos *et al.*, 2015)
AZPAE14859	2007	Spain	Bilbao	(Kos *et al.*, 2015)
AZPAE14860	2007	Spain	Bilbao	(Kos *et al.*, 2015)
AZPAE14861	2007	Spain	Bilbao	(Kos *et al.*, 2015)
AZPAE14862	2007	India	Chennai	(Kos *et al.*, 2015)
AZPAE14863	2007	India	Chennai	(Kos *et al.*, 2015)
AZPAE14864	2007	India	Chennai	(Kos *et al.*, 2015)
AZPAE14865	2007	India	Chennai	(Kos *et al.*, 2015)

Continued on next page

Table S1 – continued from previous page

Isolat	Year of isolation	Country	City	Reference
AZPAE14866	2007	China	Shatin	(Kos *et al.*, 2015)
AZPAE14867	2007	China	Shatin	(Kos *et al.*, 2015)
AZPAE14868	2007	Argentina	Victoria	(Kos *et al.*, 2015)
AZPAE14869	2007	Argentina	Victoria	(Kos *et al.*, 2015)
AZPAE14870	2007	Argentina	Victoria	(Kos *et al.*, 2015)
AZPAE14871	2007	Argentina	Victoria	(Kos *et al.*, 2015)
AZPAE14872	2007	Argentina	Victoria	(Kos *et al.*, 2015)
AZPAE14873	2007	Argentina	Victoria	(Kos *et al.*, 2015)
AZPAE14874	2007	United States	Detroit	(Kos *et al.*, 2015)
AZPAE14875	2007	United States	Detroit	(Kos *et al.*, 2015)
AZPAE14876	2007	United States	Detroit	(Kos *et al.*, 2015)
AZPAE14877	2007	United States	Roseburg	(Kos *et al.*, 2015)
AZPAE14878	2007	United States	Roseburg	(Kos *et al.*, 2015)
AZPAE14879	2007	United States	Roseburg	(Kos *et al.*, 2015)
AZPAE14880	2007	Spain	Santander	(Kos *et al.*, 2015)
AZPAE14881	2007	Spain	Santander	(Kos *et al.*, 2015)
AZPAE14882	2007	Spain	Santander	(Kos *et al.*, 2015)
AZPAE14883	2007	Croatia	Split	(Kos *et al.*, 2015)
AZPAE14884	2007	Croatia	Split	(Kos *et al.*, 2015)
AZPAE14885	2007	Croatia	Split	(Kos *et al.*, 2015)
AZPAE14886	2007	Croatia	Split	(Kos *et al.*, 2015)
AZPAE14887	2007	Croatia	Split	(Kos *et al.*, 2015)
AZPAE14888	2007	United States	Detroit	(Kos *et al.*, 2015)
AZPAE14889	2008	China	Shatin	(Kos *et al.*, 2015)
AZPAE14890	2008	France	Besancon	(Kos *et al.*, 2015)
AZPAE14891	2008	France	Besancon	(Kos *et al.*, 2015)
AZPAE14892	2008	France	Besancon	(Kos *et al.*, 2015)
AZPAE14893	2008	France	Besancon	(Kos *et al.*, 2015)
AZPAE14894	2008	Germany	Heidelberg	(Kos *et al.*, 2015)
AZPAE14895	2008	Germany	Heidelberg	(Kos *et al.*, 2015)
AZPAE14897	2008	India	Chennai	(Kos *et al.*, 2015)
AZPAE14898	2008	India	Chennai	(Kos *et al.*, 2015)
AZPAE14899	2008	India	Chennai	(Kos *et al.*, 2015)
AZPAE14900	2008	India	Chennai	(Kos *et al.*, 2015)
AZPAE14901	2008	India	Chennai	(Kos *et al.*, 2015)
AZPAE14902	2008	Argentina	Victoria	(Kos *et al.*, 2015)
AZPAE14903	2008	Spain	Madrid	(Kos *et al.*, 2015)
AZPAE14904	2008	Spain	Madrid	(Kos *et al.*, 2015)

Continued on next page

Table S1 – continued from previous page

Isolat	Year of isolation	Country	City	Reference
AZPAE14905	2008	Germany	Koln	(Kos *et al.*, 2015)
AZPAE14906	2008	Germany	Koln	(Kos *et al.*, 2015)
AZPAE14907	2008	China	Shatin	(Kos *et al.*, 2015)
AZPAE14908	2008	Spain	Palma de Mallorca	(Kos *et al.*, 2015)
AZPAE14909	2008	Spain	Palma de Mallorca	(Kos *et al.*, 2015)
AZPAE14910	2008	India	Mumbai	(Kos *et al.*, 2015)
AZPAE14911	2008	India	Mumbai	(Kos *et al.*, 2015)
AZPAE14912	2008	Croatia	Split	(Kos *et al.*, 2015)
AZPAE14913	2008	Croatia	Split	(Kos *et al.*, 2015)
AZPAE14914	2008	Spain	Santander	(Kos *et al.*, 2015)
AZPAE14915	2008	Spain	Santander	(Kos *et al.*, 2015)
AZPAE14916	2008	Spain	Bilbao	(Kos *et al.*, 2015)
AZPAE14917	2008	Spain	Bilbao	(Kos *et al.*, 2015)
AZPAE14918	2008	Spain	Bilbao	(Kos *et al.*, 2015)
AZPAE14919	2008	Spain	Bilbao	(Kos *et al.*, 2015)
AZPAE14920	2008	Spain	Bilbao	(Kos *et al.*, 2015)
AZPAE14921	2009	France	Paris	(Kos *et al.*, 2015)
AZPAE14922	2009	France	Paris	(Kos *et al.*, 2015)
AZPAE14923	2008	Brazil	Sao Paulo	(Kos *et al.*, 2015)
AZPAE14924	2008	Brazil	Sao Paulo	(Kos *et al.*, 2015)
AZPAE14925	2008	Brazil	Sao Paulo	(Kos *et al.*, 2015)
AZPAE14926	2008	Brazil	Sao Paulo	(Kos *et al.*, 2015)
AZPAE14927	2008	Brazil	Sao Paulo	(Kos *et al.*, 2015)
AZPAE14928	2008	Brazil	Sao Paulo	(Kos *et al.*, 2015)
AZPAE14929	2009	Germany	Aachen	(Kos *et al.*, 2015)
AZPAE14930	2009	Germany	Aachen	(Kos *et al.*, 2015)
AZPAE14931	2009	Germany	Aachen	(Kos *et al.*, 2015)
AZPAE14932	2009	Germany	Aachen	(Kos *et al.*, 2015)
AZPAE14933	2009	France	Rouen	(Kos *et al.*, 2015)
AZPAE14934	2009	France	Rouen	(Kos *et al.*, 2015)
AZPAE14935	2008	France	Rouen	(Kos *et al.*, 2015)
AZPAE14936	2008	Brazil	Curitiba	(Kos *et al.*, 2015)
AZPAE14937	2008	France	Rouen	(Kos *et al.*, 2015)
AZPAE14938	2008	France	Rouen	(Kos *et al.*, 2015)
AZPAE14939	2009	Colombia	Bogota	(Kos *et al.*, 2015)
AZPAE14940	2009	France	Rouen	(Kos *et al.*, 2015)
AZPAE14941	2009	China	Hong Kong	(Kos *et al.*, 2015)
AZPAE14942	2009	China	Hong Kong	(Kos *et al.*, 2015)

Continued on next page

Table S1 – continued from previous page

Isolat	Year of isolation	Country	City	Reference
AZPAE14943	2009	Colombia	Bogota	(Kos *et al.*, 2015)
AZPAE14944	2009	United States	Detroit	(Kos *et al.*, 2015)
AZPAE14945	2009	United States	Detroit	(Kos *et al.*, 2015)
AZPAE14946	2009	United States	Detroit	(Kos *et al.*, 2015)
AZPAE14947	2009	China	Beijing	(Kos *et al.*, 2015)
AZPAE14948	2009	Argentina	Victoria	(Kos *et al.*, 2015)
AZPAE14949	2009	Argentina	Victoria	(Kos *et al.*, 2015)
AZPAE14950	2009	Argentina	Victoria	(Kos *et al.*, 2015)
AZPAE14951	2009	Argentina	Victoria	(Kos *et al.*, 2015)
AZPAE14952	2009	China	Shatin	(Kos *et al.*, 2015)
AZPAE14953	2009	China	Shatin	(Kos *et al.*, 2015)
AZPAE14954	2009	France	Nantes	(Kos *et al.*, 2015)
AZPAE14955	2009	France	Nantes	(Kos *et al.*, 2015)
AZPAE14956	2009	Germany	München	(Kos *et al.*, 2015)
AZPAE14957	2009	Germany	München	(Kos *et al.*, 2015)
AZPAE14958	2009	India	Mumbai	(Kos *et al.*, 2015)
AZPAE14959	2009	India	Mumbai	(Kos *et al.*, 2015)
AZPAE14960	2009	Spain	Palma de Mallorca	(Kos *et al.*, 2015)
AZPAE14961	2009	Spain	Palma de Mallorca	(Kos *et al.*, 2015)
AZPAE14962	2009	Spain	Palma de Mallorca	(Kos *et al.*, 2015)
AZPAE14963	2009	Spain	Palma de Mallorca	(Kos *et al.*, 2015)
AZPAE14964	2009	France	Nantes	(Kos *et al.*, 2015)
AZPAE14965	2009	France	Nantes	(Kos *et al.*, 2015)
AZPAE14967	2009	Croatia	Split	(Kos *et al.*, 2015)
AZPAE14968	2009	Croatia	Split	(Kos *et al.*, 2015)
AZPAE14969	2010	United States	Roseburg	(Kos *et al.*, 2015)
AZPAE14970	2010	United States	Roseburg	(Kos *et al.*, 2015)
AZPAE14971	2010	China	Shatin	(Kos *et al.*, 2015)
AZPAE14972	2010	Germany	Aachen	(Kos *et al.*, 2015)
AZPAE14973	2010	Germany	Aachen	(Kos *et al.*, 2015)
AZPAE14974	2010	Germany	Aachen	(Kos *et al.*, 2015)
AZPAE14975	2010	China	Beijing	(Kos *et al.*, 2015)
AZPAE14976	2010	China	Beijing	(Kos *et al.*, 2015)
AZPAE14977	2010	China	Beijing	(Kos *et al.*, 2015)
AZPAE14978	2010	United States	Roseburg	(Kos *et al.*, 2015)
AZPAE14979	2010	United States	Roseburg	(Kos *et al.*, 2015)
AZPAE14980	2010	United States	Detroit	(Kos *et al.*, 2015)
AZPAE14981	2010	France	Paris	(Kos *et al.*, 2015)

Continued on next page

Table S1 – continued from previous page

Isolat	Year of isolation	Country	City	Reference
AZPAE14982	2010	Croatia	Split	(Kos *et al.*, 2015)
AZPAE14983	2010	Croatia	Split	(Kos *et al.*, 2015)
AZPAE14984	2010	France	Paris	(Kos *et al.*, 2015)
AZPAE14985	2010	China	Shatin	(Kos *et al.*, 2015)
AZPAE14986	2010	China	Shatin	(Kos *et al.*, 2015)
AZPAE14987	2010	Germany	Koln	(Kos *et al.*, 2015)
AZPAE14988	2010	China	Shatin	(Kos *et al.*, 2015)
AZPAE14989	2010	China	Hong Kong	(Kos *et al.*, 2015)
AZPAE14990	2010	China	Hong Kong	(Kos *et al.*, 2015)
AZPAE14991	2010	Spain	Santander	(Kos *et al.*, 2015)
AZPAE14992	2010	Germany	München	(Kos *et al.*, 2015)
AZPAE14993	2010	Spain	Madrid	(Kos *et al.*, 2015)
AZPAE14994	2010	Germany	Heidelberg	(Kos *et al.*, 2015)
AZPAE14995	2010	Spain	Palma de Mallorca	(Kos *et al.*, 2015)
AZPAE14996	2010	Spain	Palma de Mallorca	(Kos *et al.*, 2015)
AZPAE14997	2010	Spain	Palma de Mallorca	(Kos *et al.*, 2015)
AZPAE14998	2010	Spain	Palma de Mallorca	(Kos *et al.*, 2015)
AZPAE14999	2010	Spain	Bilbao	(Kos *et al.*, 2015)
AZPAE15000	2010	Spain	Bilbao	(Kos *et al.*, 2015)
AZPAE15001	2011	Colombia	Bogota	(Kos *et al.*, 2015)
AZPAE15002	2010	Spain	Bilbao	(Kos *et al.*, 2015)
AZPAE15003	2011	Colombia	Bogota	(Kos *et al.*, 2015)
AZPAE15004	2011	Colombia	Bogota	(Kos *et al.*, 2015)
AZPAE15005	2011	United States	Roseburg	(Kos *et al.*, 2015)
AZPAE15006	2011	United States	Roseburg	(Kos *et al.*, 2015)
AZPAE15007	2010	Spain	Santander	(Kos *et al.*, 2015)
AZPAE15008	2010	Spain	Santander	(Kos *et al.*, 2015)
AZPAE15009	2010	Spain	Santander	(Kos *et al.*, 2015)
AZPAE15010	2010	Spain	Santander	(Kos *et al.*, 2015)
AZPAE15011	2010	Spain	Santander	(Kos *et al.*, 2015)
AZPAE15012	2011	Germany	Koln	(Kos *et al.*, 2015)
AZPAE15013	2011	Germany	Koln	(Kos *et al.*, 2015)
AZPAE15014	2011	Germany	Koln	(Kos *et al.*, 2015)
AZPAE15015	2011	Germany	Koln	(Kos *et al.*, 2015)
AZPAE15016	2011	United States	Detroit	(Kos *et al.*, 2015)
AZPAE15017	2011	United States	Detroit	(Kos *et al.*, 2015)
AZPAE15018	2011	United States	Detroit	(Kos *et al.*, 2015)
AZPAE15019	2010	France	Besancon	(Kos *et al.*, 2015)

Continued on next page

Table S1 – continued from previous page

Isolat	Year of isolation	Country	City	Reference
AZPAE15020	2010	France	Besancon	(Kos *et al.*, 2015)
AZPAE15021	2011	Argentina	Victoria	(Kos *et al.*, 2015)
AZPAE15022	2011	France	Nantes	(Kos *et al.*, 2015)
AZPAE15023	2011	Spain	Madrid	(Kos *et al.*, 2015)
AZPAE15024	2011	Spain	Madrid	(Kos *et al.*, 2015)
AZPAE15025	2011	Spain	Madrid	(Kos *et al.*, 2015)
AZPAE15026	2011	Colombia	Bogota	(Kos *et al.*, 2015)
AZPAE15027	2011	Spain	Bilbao	(Kos *et al.*, 2015)
AZPAE15028	2011	France	Paris	(Kos *et al.*, 2015)
AZPAE15029	2011	France	Paris	(Kos *et al.*, 2015)
AZPAE15030	2011	Germany	München	(Kos *et al.*, 2015)
AZPAE15031	2011	Germany	Aachen	(Kos *et al.*, 2015)
AZPAE15032	2011	France	Rouen	(Kos *et al.*, 2015)
AZPAE15033	2011	France	Rouen	(Kos *et al.*, 2015)
AZPAE15034	2011	Spain	Bilbao	(Kos *et al.*, 2015)
AZPAE15035	2011	Spain	Bilbao	(Kos *et al.*, 2015)
AZPAE15036	2012	China	Shatin	(Kos *et al.*, 2015)
AZPAE15037	2012	France	Paris	(Kos *et al.*, 2015)
AZPAE15038	2012	France	Paris	(Kos *et al.*, 2015)
AZPAE15039	2012	Germany	Heidelberg	(Kos *et al.*, 2015)
AZPAE15040	2012	Germany	Heidelberg	(Kos *et al.*, 2015)
AZPAE15041	2012	Germany	Koln	(Kos *et al.*, 2015)
AZPAE15042	2012	Germany	Heidelberg	(Kos *et al.*, 2015)
AZPAE15043	2012	France	Nantes	(Kos *et al.*, 2015)
AZPAE15044	2012	France	Nantes	(Kos *et al.*, 2015)
AZPAE15045	2011	France	Nantes	(Kos *et al.*, 2015)
AZPAE15046	2012	Argentina	Victoria	(Kos *et al.*, 2015)
AZPAE15047	2012	Argentina	Victoria	(Kos *et al.*, 2015)
AZPAE15048	2012	Germany	München	(Kos *et al.*, 2015)
AZPAE15049	2012	Germany	München	(Kos *et al.*, 2015)
AZPAE15050	2012	China	Hong Kong	(Kos *et al.*, 2015)
AZPAE15051	2012	China	Hong Kong	(Kos *et al.*, 2015)
AZPAE15052	2012	Argentina	Victoria	(Kos *et al.*, 2015)
AZPAE15053	2012	Argentina	Victoria	(Kos *et al.*, 2015)
AZPAE15054	2012	Colombia	Bogota	(Kos *et al.*, 2015)
AZPAE15055	2012	Colombia	Bogota	(Kos *et al.*, 2015)
AZPAE15056	2012	China	Beijing	(Kos *et al.*, 2015)
AZPAE15057	2012	China	Beijing	(Kos *et al.*, 2015)

Continued on next page

Table S1 – continued from previous page

Isolat	Year of isolation	Country	City	Reference
AZPAE15058	2012	France	Nantes	(Kos *et al.*, 2015)
AZPAE15059	2012	France	Nantes	(Kos *et al.*, 2015)
AZPAE15060	2012	France	Nantes	(Kos *et al.*, 2015)
AZPAE15061	2012	France	Nantes	(Kos *et al.*, 2015)
AZPAE15062	2012	Brazil	Curitiba	(Kos *et al.*, 2015)
AZPAE15063	2012	Brazil	Curitiba	(Kos *et al.*, 2015)
AZPAE15064	2012	Brazil	Curitiba	(Kos *et al.*, 2015)
AZPAE15065	2012	Brazil	Curitiba	(Kos *et al.*, 2015)
AZPAE15066	2003	Croatia	Split	(Kos *et al.*, 2015)
AZPAE15067	2004	Germany	Heidelberg	(Kos *et al.*, 2015)
AZPAE15068	2004	Germany	Heidelberg	(Kos *et al.*, 2015)
AZPAE15069	2004	Germany	Heidelberg	(Kos *et al.*, 2015)
AZPAE15070	2004	Germany	Heidelberg	(Kos *et al.*, 2015)
AZPAE15071	2004	Germany	Heidelberg	(Kos *et al.*, 2015)
AZPAE15072	2004	Germany	Heidelberg	(Kos *et al.*, 2015)
PAO1	1954	Australia	Melbourne	(Holloway, 1955)
PA14	Unknown	Unknown	Unknown	(Rahme *et al.*, 1995)
PA7	Unknown	Argentina	Unknown	(Roy *et al.*, 2010)

Table S2. Primer sequences used to construct mutant genotypes. The forward inner primers (those named with inner For) include a single capital letter (in bold) which coded for the site directed mutation of interest. Lower case letters represent sequence homology or spacers, the un-bolded capital letters represent restriction enzyme (Res. Enz.) recognition sequence.

Name	Primer Sequence	Res. Enz.
gyrA_inner_For	gcgGGTCTCgcgaca**T**cgcggtctacgacaccatc	BsaI
gyrA_outer_Rev	gcgGGTCTCAGCAAgccaccacgttgatgccg	BsaI
gyrA_inner_Rev	gcgGGTCTCtgtcgccgtgcgggtgg	BsaI
gyrA_outer_For	gcgGGTCTCAGTCGgcgaggacatcccgatcgaag	BsaI
parC_outer_For	gcgGAAGACaCAATTGACTAGTatcatcccctaaccagcgcc	BbsI, MfeI, SpeI
parC_inner_Rev	gcgGAAGACcggcctgctacgaggcc	BbsI
parC_S87L_inner_For	gcgGAAGACgcaggcc**A**agtcgccgtgcgggtgg	BbsI
parC_S87W_inner_For	gcgGAAGACgcaggcc**C**agtcgccgtgcgggtgg	BbsI
parC_outer_Rev	gcgGAAGACGGCGCGCCtactacgccctcgacgaagc	BbsI, AscI

Table S3. Correlation between know resistance determining loci and resistance phenotype. With knowledge of the resistance phenotype for strains in our alignment, we counted which strains had mutations in the codon of amino acid (AA) positions known to confer fluoroquinolone resistance. We used a chi-squared test for the independence between mutation at these positions and the resistance phenotype and highlight significant ($P \leq 0.05$) values in bold. Recall there were 389 strains in our alignment of which 192 (197) were susceptible (resistant).

Gene	AA	Mutants	Resistant	X^2	df	P
gyrA	83	161	156	231.941	1	**2.250** $\times 10^{-52}$
	87	34	33	30.110	1	**4.083** $\times 10^{-08}$
gyrB	466	16	14	7.596	1	**0.006**
	468	9	9	7.072	1	**0.008**
parC	87	128	127	177.208	1	**1.979** $\times 10^{-40}$
	91	6	6	4.103	1	**0.043**
parE	457	8	7	3.061	1	0.080
	459	4	3	0.227	1	0.634
	473	11	7	0.323	1	0.570
morA	563	14	7	0.000	1	1.000
	975	15	6	0.333	1	0.564
	1056	15	6	0.333	1	0.564
	1109	15	7	0.003	1	0.960
	1155	16	9	0.041	1	0.839
	1162	15	8	0.000	1	1.000
	1213	15	7	0.003	1	0.960

Table S4. Evidence of relative ΔG epistasis among the strongly correlated pairs of synonymous intragenic substitutions. Epistasis is measured with a multiplicative model and error is calculated using error propagation (Trindade *et al.*, 2009). There is evidence for epistasis when the absolute value of is greater than the error of our measures. We find no evidence for epistasis as ϵ is never greater than estimates of error.

**Mutant Pair**		ϵ	**Error**
dnaN c495t	*dnaN* c504t	-9.263 $\times 10^{-4}$	2.387 $\times 10^{-2}$
gyrB c1422t	*gyrB* c1443t	-1.481 $\times 10^{-4}$	4.768 $\times 10^{-3}$
morA a4041g	*morA* c4083t	1.113 $\times 10^{-2}$	3.394 $\times 10^{-2}$
parC c1533t	*parC* c1581t	-7.982 $\times 10^{-3}$	2.794 $\times 10^{-2}$
parC c1533t	*parC* c1587t	8.039 $\times 10^{-4}$	2.514 $\times 10^{-2}$
parC c1533t	*parC* t1554g	-1.798 $\times 10^{-2}$	3.166 $\times 10^{-2}$
parC c1581t	*parC* c1587t	4.633 $\times 10^{-4}$	2.635 $\times 10^{-2}$
parC t1554g	*parC* c1581t	-6.985 $\times 10^{-3}$	3.529 $\times 10^{-2}$

Table S5. Evidence of I_{TE} epistasis among the strongly correlated pairs of synonymous intragenic substitutions. Epistasis is measured with a multiplicative model and error is calculated using error propagation (Trindade *et al.*, 2009). There is evidence for epistasis when the absolute value of is greater than the error of our measures. We find negative epistasis for the sites in *morA* based on ϵ having an absolute value greater than the estimates of error.

**Mutational Pair**		ϵ	**Error**
dnaN c504t	*dnaN* c495t	1.4113×10^{-5}	3.234×10^{-3}
gyrB c1443t	*gyrB* c1422t	5.330×10^{-5}	1.334×10^{-2}
morA c4083t	*morA* a4041g	$\mathbf{-4.437} \times 10^{-2}$	$\mathbf{1.053} \times 10^{-2}$
parC c1581t	*parC* c1587t	1.390×10^{-5}	4.606×10^{-3}
parC c1533t	*parC* c1587t	1.362×10^{-5}	7.304×10^{-3}
parC t1554g	*parC* c1581t	1.769×10^{-6}	2.770×10^{-3}
parC c1533t	*parC* c1581t	3.148×10^{-5}	6.041×10^{-3}
parC c1533t	*parC* t1554g	-1.025×10^{-5}	5.360×10^{-3}

Table S6. Test for independence between the relative position of correlated substitutions and the type of pair they form. We used a χ^2 test and present the observed (expected) counts for the number of significantly correlated pairs ($P \leq 10^{-4}$) of substitutions based on if they were in the same gene and how many of the substitutions were synonymous. Analysis led to a test statistic of 7246.04 with 2 degrees of freedom and $P \sim 0$.

**Within the same gene**	**Type of Pair**		
	**Non Synonymous**	**One Synonymous**	**Synonymous**
**TRUE**	0 (0.52)	6 (1.83)	60 (1.63)
**FALSE**	101 (1289.31)	2274 (4569.33)	7470 (4048.38)

Table S7. Absorbance (600 nm) readings from ciprofloxacin MIC assay of *P. aeruginosa* WT and mutant constructs. Readings were taken after 24 hours of growth on a 96 well plate where wells contained half salt LB growth medium with ciprofloxacin concentration as denoted by the column heading. The values presented are the mean (standard deviation) values for a minimum of 4 replicates. No genotype appeared more than once on a single 96 well plate.

		Concentration of Ciprofloxacin ($Log_2\ \mu g/mL$)											
Background	Mutation	5	4	3	2	1	0	-1	-2	-3	-4	-5	-6
PA01	WT	0.01	0.021	0.02	0.012	0.127	0.021	0.04	0.326	0.417	0.536	0.675	0.916
		(0.006)	(0.004)	(0.002)	(0.001)	(0.156)	(0.004)	(0.02)	(0.034)	(0.001)	(0.074)	(0.273)	(0.006)
	gyrA c248t	0.013	0.017	0.1	0.398	0.521	0.512	0.754	0.764	0.772	0.779	0.814	0.912
		(0.004)	(0.007)	(0.042)	(0.149)	(0.087)	(0.029)	(0.134)	(0.087)	(0.078)	(0.091)	(0.068)	(0.09)
	parC c260t	0.016	0.031	0.036	0.014	0.053	0.034	0.059	0.447	0.468	0.504	0.639	0.894
		(0.003)	(0.017)	(0.034)	(0.006)	(0.057)	(0.025)	(0.019)	(0.03)	(0.037)	(0.052)	(0.06)	(0.122)
	parC c260g	0.015	0.034	0.022	0.018	0.014	0.029	0.098	0.422	0.463	0.499	0.698	0.859
		(0.005)	(0.018)	(0.006)	(0.001)	(0.002)	(0.019)	(0.114)	(0.057)	(0.053)	(0.06)	(0.063)	(0.129)
	gyrA c248t,	0.418	0.76	0.843	0.803	0.845	0.805	0.754	0.748	0.686	0.807	0.774	0.854
	parC c260t	(0.116)	(0.094)	(0.138)	(0.113)	(0.172)	(0.13)	(0.145)	(0.138)	(0.138)	(0.085)	(0.091)	(0.139)
	gyrA c248t,	0.376	0.598	0.629	0.768	0.814	0.836	0.918	0.81	0.826	0.854	0.881	0.982
	parC c260g	(0.386)	(0.179)	(0.116)	(0.132)	(0.057)	(0.07)	(0.059)	(0.099)	(0.111)	(0.038)	(0.033)	(0.071)
PA14	WT	0.044	0.054	0.063	0.084	0.094	0.1	0.02	0.02	0.04	0.285	0.615	0.936
		(0.006)	(0.005)	(0.01)	(0.012)	(0.017)	(0.093)	(0.004)	(0.003)	(0.019)	(0.22)	(0.163)	(0.086)
	gyrA c248t	0.07	0.079	0.087	0.314	0.25	0.133	0.264	0.864	0.895	0.952	0.899	1.096
		(0.013)	(0.005)	(0.036)	(0.071)	(0.034)	(0.05)	(0.145)	(0.122)	(0.054)	(0.059)	(0.065)	(0.035)
	parC c260t	0.04	0.064	0.096	0.12	0.114	0.07	0.022	0.033	0.025	0.226	0.659	0.819
		(0.007)	(0.004)	(0.008)	(0.028)	(0.018)	(0.013)	(0.003)	(0.013)	(0.003)	(0.085)	(0.119)	(0.169)
	parC c260g	0.045	0.095	0.1	0.13	0.121	0.07	0.028	0.04	0.03	0.249	0.649	0.961
		(0.004)	(0.025)	(0.058)	(0.06)	(0.047)	(0.04)	(0.006)	(0.003)	(0.004)	(0.086)	(0.1)	(0.145)
	gyrA c248t,	0.279	0.294	0.237	0.769	0.996	0.957	0.941	0.956	0.933	0.948	0.927	0.941
	parC c260t	(0.012)	(0.029)	(0.038)	(0.184)	(0.077)	(0.056)	(0.019)	(0.018)	(0.027)	(0.028)	(0.018)	(0.012)
	gyrA c248t,	0.195	0.25	0.268	0.809	1.054	0.956	0.92	0.926	0.921	0.921	0.932	0.987
	parC c260g	(0.078)	(0.041)	(0.096)	(0.064)	(0.073)	(0.048)	(0.056)	(0.022)	(0.035)	(0.025)	(0.038)	(0.125)

SI Figures

Figure S1. Comparisons of observed and expected types of correlated substitutions with at least weak ($P \leq 10^{-4}$) support. Note that all y-axes are based on $\log_2$ counts and so when there is no bar the count was 0. Panels a,c,e show the observed distributions of when correlated pairs were: (a) in genes we expected to show correlation, (c) nonsynonymous, or (e) intragenic. Whereas b,d,f show the null expected distributions, of their left-most counterpart, if a same number of correlated pairs had been randomly drawn from our alignment.

Figure S2. Phylogenetic trees estimated from the concatenated alignment of all conserved Information class, determined from COG terms, genes in our dataset. Support for bifurcations are printed at nodes and were estimated using FastTree's default SH test for local support values. (a) shows the unrooted tree which includes the taxonomic outliers identified by being part of the long branch sub-clade including PA7. (b) shows the rooted tree once the PA7 sub-clade was removed. Analysis of correlated evolution, with AEGIS, was performed with the tree presented in (b).

Figure S3. Phylogeny of the gene(s) which contain the most significantly correlated pairs of substitutions ($P < 10^{11}$). Each tree was constructed with FastTree (Price *et al.*, 2010) (GTR $+\Gamma_5$). The title of each panel indicates the pair of substitutions being considered. For trees on the left hand side, the color of the tips represent the paired nucleotide state of strains. On the right side, the color of tips represent the I_{TE} value of the whole gene(s) sequence.

Figure S4. Top 100 ranked importance values in predicting levofloxacin (a fluoroquinolone drug) resistance for nucleotide positions in our alignment. The importance values were obtained by running the adaptive boosting machine learning algorithm *boosting* (implemented in the R package *adabag* (Alfaro *et al.*, 2013)) on all polymorphic sites in our alignment. Levofloxacin resistance phenotype information was obtained from previously published data (Kos *et al.*, 2015). Importance values reflect the strength of correlation between genomic sites and the resistance phenotype. Red circles denote genomic positions which have been previously reported in the literature to correlate with fluoroquinolone resistance.

Figure S5. The signal of correlated evolution for paired synonymous substitutions as a function of physical distance. Physical distance is measured as the number of base pairs, using the circular reference chromosome of PA14, separating two mutations. The red line shows the linear regression for all significantly correlated pairs with at least medium support ($P \leq 10^{-7}$). Purple highlights the most significantly correlated pairs of substitutions.
